# Impact of pet ownership in early childhood at ages 1 and 4–5 years on mental health at ages 7–8: findings from the INMA project

**DOI:** 10.1007/s12519-025-00942-2

**Published:** 2025-10-04

**Authors:** Llúcia González, Mònica Guxens, Blanca Sarzo, Ainara Andiarena, Loreto Santa-Marina, Adonina Tardón, Jordi Julvez, Cristina Rodríguez-Dehli, Marisa Rebagliato, Marisa Estarlich

**Affiliations:** 1 Spanish Consortium for Research on Epidemiology and Public Health (CIBERESP), Instituto de Salud Carlos III, C/Monforte de Lemos, 3-5, 28029 Madrid, Spain; 2 Joint Research Unit in Epidemiology, Environment and Health, FISABIO-UJI-UV, Valencia, Spain; 3 Department of Child and Adolescent Psychiatry/Psychology, Erasmus MC, University Medical Centre, Rotterdam, The Netherlands; 4 Universitat Pompeu Fabra, Barcelona, Spain; 5 ISGlobal, Barcelona Biomedical Research Park (PRBB), Eighty-Eighth Doctor Aiguader Av, 08003 Barcelona, Spain; 6 ICREA, Barcelona, Spain; 7 Foundation for the Promotion of Health and Biomedical Research of the Valencian Region, Valencia, Spain; 8 Faculty of Psychology, University of the Basque Country, UPV/EHU, 20018 San Sebastian, Spain; 9 Biogipuzkoa Health Research Institute, Environmental Epidemiology and Child Development Group, San Sebastian, Spain; 10 Ministry of Health of the Basque Government, Subdirectorate for Public Health and Addictions of Gipuzkoa, San Sebastian, Spain; 11 Instituto Universitario de Oncología del Principado de Asturias (IUOPA), Universidad de Oviedo, Health Research Institute of Asturias (ISPA), Oviedo, Spain; 12 Clinical and Epidemiological Neuroscience (Neuroèpia), Institut d’Investigació Sanitària Pere Virgili (IISPV), Reus, Spain; 13 Unidad de Endocrinología Pediátrica, Hospital Universitario San Agustín, Avilés, Spain; 14 Predepartamental Unit of Medicine, Health Sciences Faculty, Universitat Jaume I, Sos Baynat Av, 12600 Castelló de La Plana, Spain; 15 Nursing and Chiropody Faculty of Valencia University, Nineteenth of Menéndez Pelayo St., 46010 Valencia, Spain

**Keywords:** Cohort study, Inverse probability weighting, Mental health, Pet ownership

## Abstract

**Background:**

We aimed to explore associations between the presence of pets at one and 4–5 years of age with internalizing and externalizing problems at 7–8 years.

**Methods:**

Participants comprised 1893 families from the INfancia y Medio Ambiente (INMA) project. Information was collected on the presence of (1) any pet, (2) dogs, (3) cats, (4) birds or (5) other animals. Pet ownership was categorized as never, always, only at age 1 and only at age 4–5. Internalizing and externalizing problems were measured at ages 7–8 years through the Strengths and Difficulties Questionnaire, a Likert questionnaire on children’s behavioural and emotional symptoms. Negative binomial regression models and Tukey’s multiple comparison tests were used to analyse data sets. Five sensitivity analyses were performed.

**Results:**

Families that always owned a pet made up 24.4% of the sample. In addition, 11.5%, 4.5%, 3.8% and 17.6% of the families owned a dog, cat, bird or other animal, respectively. The median (P25–P75) for internalizing problems was 3 (1–5) and 5 (3–8) for externalizing problems. Owning a cat only at age 4–5 increased mental health problems: relative rate ratio (RRR) [95% confidence interval (CI)] 1.37 (1.05–1.79) for internalizing and 1.26 (1.02–1.56) for externalizing. Always having other animals was a protective factor for internalizing problems with an RRR of 0.80 (0.66–0.96). These associations remained after multiple comparison testing and sensitivity analyses.

**Conclusion:**

Owning a cat only at 4–5 years of age was linked to more internalizing and externalizing problems, whereas always having other animals was a protective factor against internalizing problems.

**Graphical abstract:**

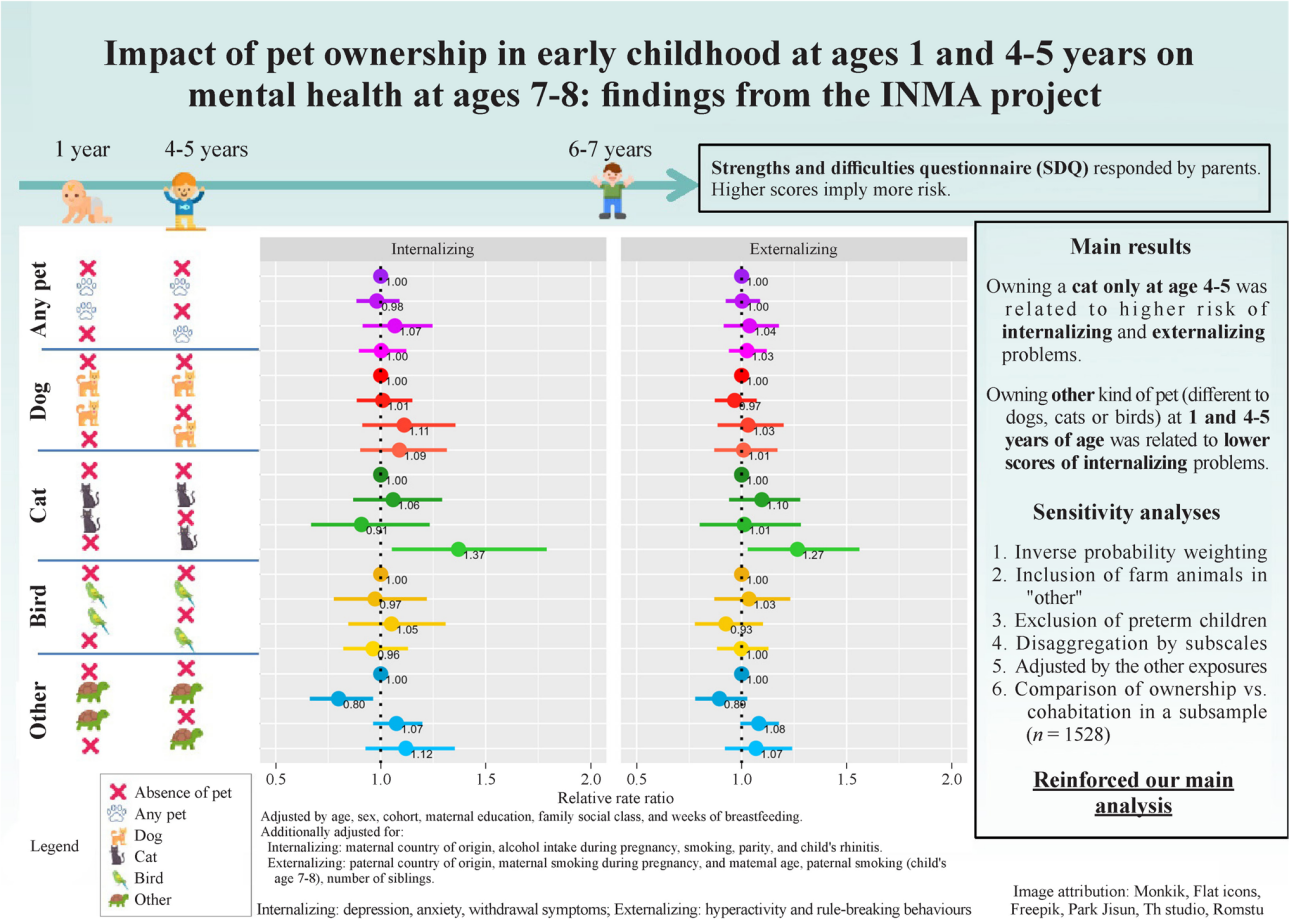

**Supplementary Information:**

The online version contains supplementary material available at 10.1007/s12519-025-00942-2.

## Introduction

Childhood represents sequential changes in cognition, behaviour and physiology from birth to adolescence [1]. Development is fostered through stable interactions in which children are involved with their family, school, peers and community, among others [2]. Attachment, defined as the emotional bond between an infant and their caregiver [3], is crucial for these social interactions, but it can also occur with animal companions [4]. Human-animal interactions during childhood enhance child development [5, 6] by fostering adaptive developmental regulation, providing emotion and cognition about animals, powering up a child’s responsibility and moral norms [6]. Pets may act as “social catalysts” to facilitate other human relationships; increase prosocial behaviour; may help children to understand nonverbal language; and finally, animals may also act as transitional objects [5].

The presence of a dog or cat may be positive for children, resulting in fewer mental health problems, such as less depression, stress, behaviour issues, and better quality of life, prosocial behaviour and well-being [7–9]. Pets provide cognitive benefits (perspective-taking abilities and intellectual development) and improve social development (social competence, social networks, social interaction and social play behaviours) [7]. They can also decrease the sense of loneliness, with this relationship being different for a family (e.g., a dog or a cat that was in the family before the child’s birth) or a child’s pet (e.g., an animal that was obtained to foster the child’s development and acquisition of responsibilities) [10]. However, some studies found inconclusive results for emotional symptoms [11] and negative outcomes for social abilities [7].

Internalizing and externalizing behaviours are a broad classification based on how children may react to stressors[12]. On the one hand, internalizing problems are more related to emotional symptoms, are inner-directed, and generate tension and suffering in the individual. They include symptoms related to depression, anxiety, somatization, etc. On the other hand, externalizing problems are behaviour problems which are outer-directed and generate discomfort in other people, and imply breaking the social rules. These problems include behaviour problems, aggressiveness, and rule-breaking behaviour, among others [13].

The majority of the studies reviewed have analysed the relationships between pet attachment and pet ownership with depression, anxiety, stress or loneliness in late childhood and adolescence. However, very few studies have explored the relationship between pets with internalizing and externalizing problems in early childhood. The Infancia y Medio Ambiente (INMA) project provides an excellent framework for exploring this relationship in the Spanish context [14]. This study aimed to analyse the effect of pet ownership in early childhood (1–5 years of age) and any consequences on child mental health at 7–8 years of age in a Spanish birth cohort.

## Methods

### Study design

The INMA project is a Spanish population-based mother–child multicentre cohort set up in 2003 in seven areas: Ribera d’Ebre, Menorca, Granada, Asturias, Valencia, Sabadell and Gipuzkoa [15]. This study uses data from the INMA Valencia, Sabadell, Asturias, and Gipuzkoa cohorts. The aim of the INMA project is to study exposure to the most important environmental pollutants in air, water and diet during pregnancy and early life and their effects on child growth and development. Over time, multiple lines of research have been developed, including the effect of pets on child development.

Recruitment procedures and inclusion criteria were described elsewhere [16]. Briefly, mothers were recruited during their first prenatal visit to their reference hospital before week 13 of gestation. The inclusion criteria were at least 16 years of age, 10–13 weeks of gestation, singleton pregnancy, intention to undergo follow-up and delivery at the corresponding reference hospital and no communication impediments.

Baseline participants were collected between November 2003–June 2005 in Valencia (*n* = 855), June 2004–September 2006 in Sabadell (*n* = 657), April 2004–June 2007 in Asturias (*n* = 494), and May 2006–February 2008 in Gipuzkoa (*n* = 638).

**Fig. 1:**
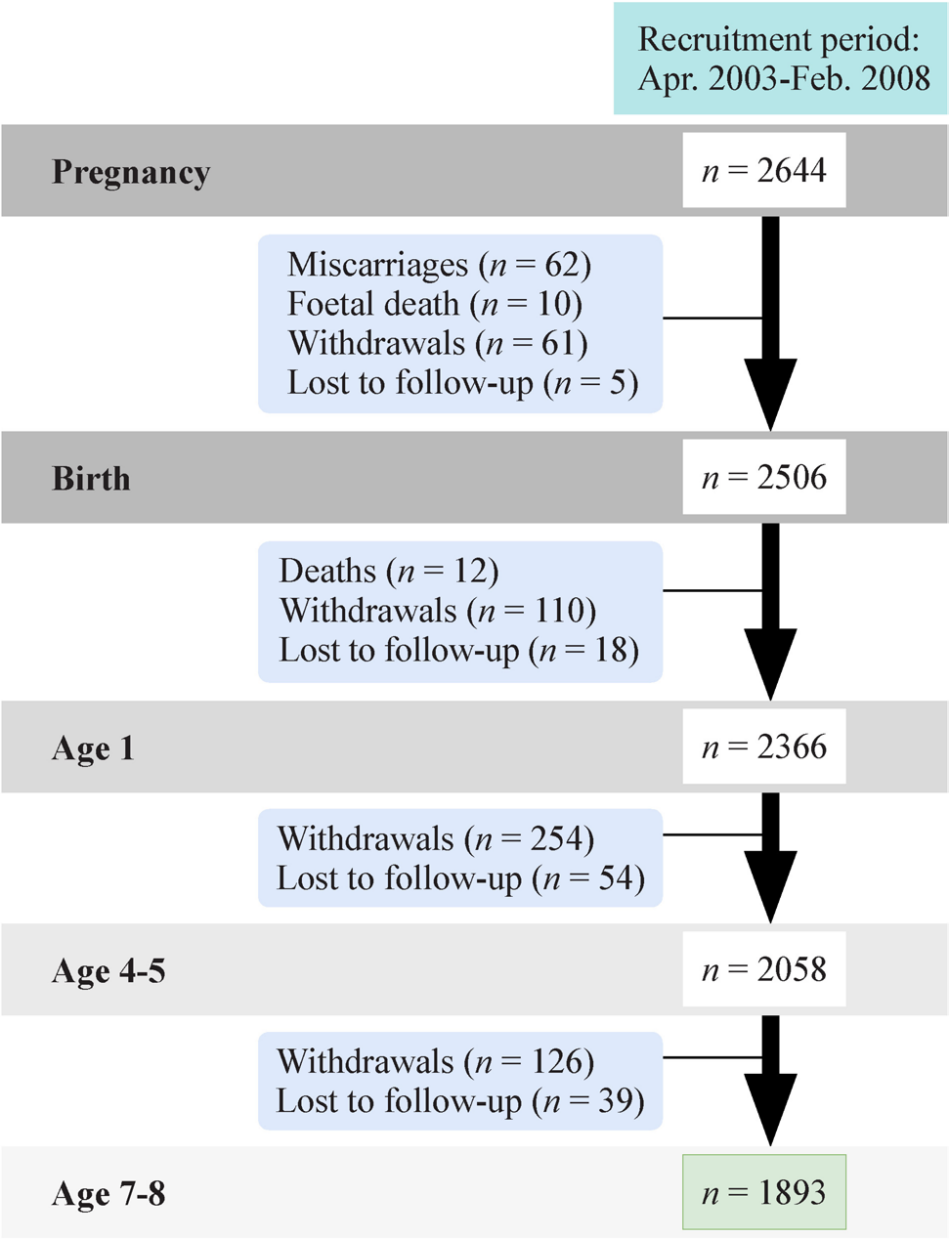
Follow-up visits and sample evolution

Follow-up visits and sample evolution are described in Fig. 1 for the joint cohorts and in Supplementary Fig. 1 for each cohort separately. Briefly, information was collected in follow-ups from pregnancy, age 1 year, age 4–5 years and age 7–8 years. The follow-up was approved by local institutional ethical review boards (Dirección General de Salud Pública; Centro Superior de Investigación en Salud Pública, Parc de Salut Mar; Regional Clinical Research Ethics Committee, Comité de Investigación del Principado de Asturias; and the Euskadi Clinical Research Ethics Committee, respectively), and the participants provided their written and informed consent to participate. This study was carried out in accordance with the principles of the Declaration of Helsinki.

### Exposure variables: pet ownership

Pet companionship was collected by an interviewer at child ages 1 and 4–5 years for five types of animals: any kind of pet, dog, cat, bird or other animal, including small mammals/rodents (rabbits, rats, hamsters, squirrels, guinea pigs), fish and reptiles/amphibia (turtles, tortoises, geckos, salamanders). For each category, presence in the previous year (no/yes) was reported. This yielded five exposure variables (any kind of pet, dog, cat, bird or other animal). A combined variable for each type of animal was created considering the two follow-ups involved, with the following categories: never/always/only at age 1/only at age 4–5 years.

### Outcome variable: mental health

Mental health in the previous six months was assessed via the Strengths and Difficulties Questionnaire (SDQ) [17]. This is a brief behavioural screening questionnaire with 25 items employed in children aged 2–17 years. It was completed by parents when the children were 7–8 years of age. The parental form presents good psychometric properties, with Chronbach’s alpha of 0.73 in the original [18] version and 0.76 in the Spanish version [19]. The SDQ comprises five subscales: (1) emotional symptoms (worry, fear, nervousness and feeling sad or unhappy); (2) conduct problems (disobedience, tantrums, fighting, lying and stealing); (3) hyperactivity/inattention (restlessness, fidgeting, distractibility, impulsivity and attention span); (4) peer problems (bullying, being picked on, having few friends or being solitary); and (5) prosocial behaviour (kind, helpful, empathic, generous) [20]. Each subscale includes five questions rated on a 3-point Likert scale [not true (0), somewhat true (1), and certainly true (2)], and each subscale can therefore be scored between 0 and 10. Higher scores indicate more behavioural problems. The prosocial subscale reflects strengths rather than difficulties, so we reversed its scoring to align the interpretation direction with the other subscales (i.e., higher scores consistently indicate greater difficulties). In this work, we employed the broadband scales of internalizing and externalizing scales as our main analysis. This can be calculated by summing subscales I + iv and ii + iii, respectively. In addition, we explored further subscales in supplementary analyses.

### Covariates

Family, parental, perinatal and child characteristics were collected through medical records and structured questionnaires at different follow-up visits (pregnancy, birth and ages 1, 4–5, and 7–8 years).

Parental sociodemographic variables were collected separately for each parent during pregnancy and consisted of occupational social class (lower/middle/upper), defined via a Spanish adaptation of the British social class classification [21] and education level (up to primary/secondary/university) defined by the International Standard Classification of Education 1997 [22]. Age, country of birth and parity were collected during pregnancy. Biological sex, preterm birth, small for gestational age and Apgar score, were obtained at birth. Type of breastfeeding and duration of exclusive breastfeeding were recorded at child’s age 1 and main care provider and nursery attendance at the child’s age 2.

Data on parental employment status were collected at the child’s age of 4–5 years. Parental intelligence was assessed with the similarity subtest of the Wechsler Adult Intelligence Scale-III [23], parental use of toxicants (tobacco and alcohol), family structure, number of siblings, type of dwelling and type of area were collected at the child’s age 4–5 years. Clinical conditions such as rhinitis or lifestyle factors such as the duration of physical activity were considered at the age of 7–8 years.

### Analyses

For descriptive analyses, frequencies and percentages were used for categorical variables, while medians and interquartile ranges were used for continuous variables. Chi-square *P* values were calculated for differences among cohorts. For bivariate analyses, we applied Mann‒Whitney *U* and Kruskal‒Wallis tests to assess possible relationships between categorical covariates and pet ownership with SDQ internalizing and externalizing scores. For continuous variables, the possible relationships between SDQ scores and covariates were assessed via Spearman’s correlations.

The relationships between pet ownership and behavioural and emotional symptoms were assessed via generalized negative binomial regression models. Cohort and child age and sex were included in all models regardless of their statistical significance. The final models were constructed following a three-step procedure. First, univariate models were implemented with the covariates and SDQ scores. Covariates significantly related to SDQ scores at *P* values < 0.20 in the likelihood ratio test were retained and included in the multivariate models. Second, multivariate models were implemented and a variable selection method was used. Thus, variables with a *P* value < 0.10 were selected, resulting in the core models. Third, the exposure variable (pet ownership) was included in each core model, yielding a total of five multivariate models for each outcome. Tukey’s multiple comparisons were applied in each model to determine differences between categories and post hoc analyses with false discovery rates were also developed to correct for multiple testing.

Six sensitivity analyses were performed: the first was to control for sample attrition using the inverse probability weighting (IPW) method [24] (Supplementary Text); the second, included farm animals in the “other animals” exposure variable; the third, excluded preterm children; the fourth analysis, disaggregated by subscale, was to check the robustness of our findings in the broadband scales (internalizing and externalizing problems). In the fifth sensitivity analysis, we adjusted each model for the remaining exposures to control for the residual effect of the ownership of other pets. The sixth analysis consisted of a comparison between pet ownership and pet cohabitation in a subsample (*n* = 1528) when these data were available (age 4–5 years).

Statistical analyses were performed using the IBM SPSS Statistics package version 26, R and RStudio (versions 4.1.3 and 2022.02.3 + 492, respectively) with the MASS, haven, foreign, ggplot2, mgcv, multcomp and sjPlot packages. Descriptive plots were developed using datawrapper [25]. Flowcharts were designed using draw.io by JGraph Ltd. [26].

## Results

### Sample description

Our sample comprised 1893 families. At the time of pregnancy, most parents belonged to the lowest social class (IV + V) (44.1%) and had achieved secondary education (41.6% mothers and 43.1% fathers) (Table 1). Most were employed (72.7% mothers and 90.5% fathers) and born in Spain. Half of the sample was primiparous. Approximately 90% of families were biparental. A regional trend was observed (Supplementary Table 2), with Gipuzkoa presenting a less deprived population. Asturias had fewer migrant parents. Biparental families were more common in Gipuzkoa and single children were more common in Asturias.

**Table 1:** Sample characteristics and their relationship to internalizing and externalizing problems

Variables	*n*	%	Internalizing				Externalizing			
			Med	P25	P75	*P*^a^	Med	P25	P75	*P*^a^
Sociodemographic characteristics										
Family social class										
Higher (I + II)	674	35.6	3.0	1.0	4.0	< 0.001	4.0	2.0	7.0	< 0.001
Middle (III)	481	25.4	2.5	1.0	4.0		5.0	3.0	7.0	
Lower (IV + V)	737	39.0	3.0	1.0	5.0		6.0	4.0	9.0	
Maternal education level										
Up to primary	377	20.0	4.0	2.0	6.0	< 0.001	6.0	4.0	9.0	< 0.001
Secondary	792	41.9	3.0	1.0	5.0		6.0	3.0	8.0	
University	719	38.1	2.0	1.0	4.0		4.0	2.0	7.0	
Paternal education level										
Up to primary	608	32.3	3.0	1.0	5.0	< 0.001	6.0	4.0	9.0	< 0.001
Secondary	834	44.3	3.0	1.0	5.0		5.0	3.0	8.0	
University	440	23.5	2.0	1.0	4.0		4.0	2.0	6.0	
Maternal employment (4–5 y)										
Working	1261	73.1	3.0	1.0	5.0	0.206	5.0	3.0	8.0	0.149
Not working	260	15.1	3.0	1.0	5.0		6.0	3.0	9.0	
Homemaker	205	11.9	3.0	1.0	4.0		5.0	3.0	7.0	
Paternal employment (4–5 y)										
Working	1548	91.1	3.0	1.0	5.0	0.004	5.0	3.0	8.0	< 0.001
Not working	151	8.9	4.0	2.0	5.0		7.0	4.0	9.0	
Maternal country of origin										
Spain	1787	94.7	3.0	1.0	5.0	0.010	5.0	3.0	8.0	0.588
Not Spain	101	5.3	4.0	2.0	6.0		5.0	3.0	8.0	
Paternal country of origin										
Spain	1766	93.4	3.0	1.0	5.0	0.060	5.0	3.0	8.0	0.494
Not Spain	124	6.6	3.5	2.0	5.0		5.0	3.0	7.0	
Parental use of tobacco and alcohol										
Maternal smoking (pregnancy)										
No	1548	83.7	3.0	1.0	5.0	< 0.001	5.0	3.0	7.0	< 0.001
Yes	302	16.3	3.0	2.0	5.0		7.0	4.0	9.0	
Paternal smoking (pregnancy)										
No	1573	84.9	3.0	1.0	5.0	0.001	5.0	3.0	7.0	0.001
Yes	280	15.1	3.0	1.0	5.0		6.0	3.0	9.0	
Maternal smoking (7–8 y)										
No	1266	74.6	3.0	1.0	5.0	< 0.001	5.0	3.0	7.0	< 0.001
Yes	430	25.4	3.0	2.0	5.0		6.0	4.0	8.0	
Paternal smoking (7–8 y)										
No	1159	69.6	3.0	1.0	5.0	0.005	5.0	3.0	7.0	< 0.001
Yes	506	30.4	3.0	1.0	5.0		6.0	3.0	9.0	
Maternal alcohol (pregnancy)										
No	1668	90.9	3.0	1.0	5.0	0.003	5.0	3.0	8.0	0.396
Yes	167	9.1	3.0	2.0	5.0		5.0	3.0	8.0	
Paternal alcohol (pregnancy)										
No	440	23.5	3.0	1.0	5.0	0.618	5.0	3.0	8.0	0.877
Yes	1429	76.5	3.0	1.0	5.0		5.0	3.0	8.0	
Family characteristics and organization										
Parity										
0	1094	57.9	3.0	1.0	5.0	< 0.001	5.0	3.0	8.0	0.155
1	689	36.4	2.0	1.0	4.0		5.0	3.0	8.0	
2 +	108	5.7	2.0	1.0	5.0		5.0	2.0	7.0	
Number of siblings (4–5 y)										
Only child	516	29.7	3.0	2.0	5.0	< 0.001	6.0	3.0	8.0	0.002
1 sibling	1063	61.1	3.0	1.0	5.0		5.0	3.0	7.0	
2 or more	160	9.2	3.0	1.0	4.0		5.0	2.0	8.0	
Mother living with (7–8 y)										
Father	1497	89.0	3.0	1.0	5.0	0.008	5.0	3.0	8.0	0.012
Other partner	94	5.6	3.0	2.0	5.0		6.0	4.0	9.0	
No partner	81	4.8	3.0	2.0	6.0		6.0	3.0	9.0	
Nursery before 24 mon										
Grandparents and other	10	0.6	4.0	2.0	5.0		6.0	6.0	10.0	
No	689	39.6	3.0	1.0	5.0	0.371	5.0	3.0	7.0	0.241
Yes	1052	60.4	3.0	1.0	5.0		5.0	3.0	8.0	
Family characteristics and organization										
Both parents are the main carers (4–5 y)										
No	1224	69.7	3.0	1.0	5.0	0.008	5.0	3.0	8.0	0.003
Yes	531	30.3	3.0	1.0	4.0		5.0	3.0	7.0	
Type of dwelling (4–5 y)										
House	151	9.4	2.0	1.0	4.0	0.470	5.0	3.0	8.0	
Terraced	214	13.3	3.0	1.0	5.0		5.0	2.0	7.0	
Flat	1240	77.0	3.0	1.0	5.0		5.0	3.0	8.0	
Other	6	0.4	3.0	2.0	6.0		8.0	3.0	9.0	0.337
Type of zone (4–5 y)										
Non-rural	1706	94.0	3.0	1.0	5.0	0.284	5.0	3.0	8.0	0.782
Rural	109	6.0	3.0	2.0	5.0		5.0	4.0	8.0	
Clinical factors										
Small for gestational age for weight (birth)										
No	1680	90.2	3.0	1.0	5.0	0.148	5.0	3.0	8.0	< 0.001
Yes	182	9.8	3.0	1.0	4.0		5.0	3.0	7.0	
Small for gestational age for head circumference (birth)										
No	1632	90.0	3.0	1.0	5.0	0.038	5.0	3.0	8.0	0.010
Yes	181	10.0	3.0	2.0	5.0		6.0	4.0	9.0	
Preterm (birth)										
No	1797	95.8	3.0	1.0	5.0	0.979	5.0	3.0	8.0	0.931
Yes	79	4.2	3.0	2.0	5.0		6.0	3.0	9.0	
Child's rhinitis (blocked nose) (4–5 y)										
No	1314	77.6	3.0	1.0	5.0	< 0.001	5.0	3.0	8.0	0.805
Yes	380	22.4	3.0	2.0	5.0		5.0	3.0	8.0	
Diagnosed with having rhinitis (4–5 y)										
No	1621	97.4	3.0	1.0	5.0	0.055	5.0	3.0	8.0	0.989
Yes	44	2.6	3.0	2.0	6.0		5.0	3.0	8.0	
Sex of the child (birth)										
Female	920	48.6	3.0	1.0	5.0	0.087	5.0	2.0	7.0	< 0.001
Male	973	51.4	3.0	1.0	5.0		6.0	4.0	9.0	

Continuous variables	Med	P25	P75	Internalizing		Externalizing				
				Coefficient	*P*^b^	Coefficient	*P*^b^			
Maternal age (pregnancy)	31.0	28.0	34.0	– 0.093	< 0.001	– 0.083	0.001			
Paternal age (pregnancy)	33.0	30.0	36.0	– 0.066	0.006	– 0.059	0.014			
Child's age	7.7	7.2	7.9	0.007	0.760	– 0.025	0.288			
Apgar 1 min (birth)	9.0	9.0	9.0	– 0.002	0.940	0.012	0.613			
Weeks of breastfeeding	21.9	6.4	39.0	– 0.063	0.009	– 0.115	< 0.001			
Maternal intelligence (WAIS) (4–5 y)	98.8	91.5	109.8	– 0.101	< 0.001	– 0.138	< 0.001			
Total extracurricular physical activity (h/d) (7–8 y)	1.0	1.0	2.0	– 0.005	0.830	0.061	0.011			

*Med* median, *P25* percentile 25, *P75* percentile 75, *WAIS* Wechsler Adult Intelligence Scale-III. ^a^*P* values of Kruskal‒Wallis test for differences between groups; ^b^*P* values of Spearman’s test

### Descriptive analysis of pet ownership and mental health

**Fig. 2:**
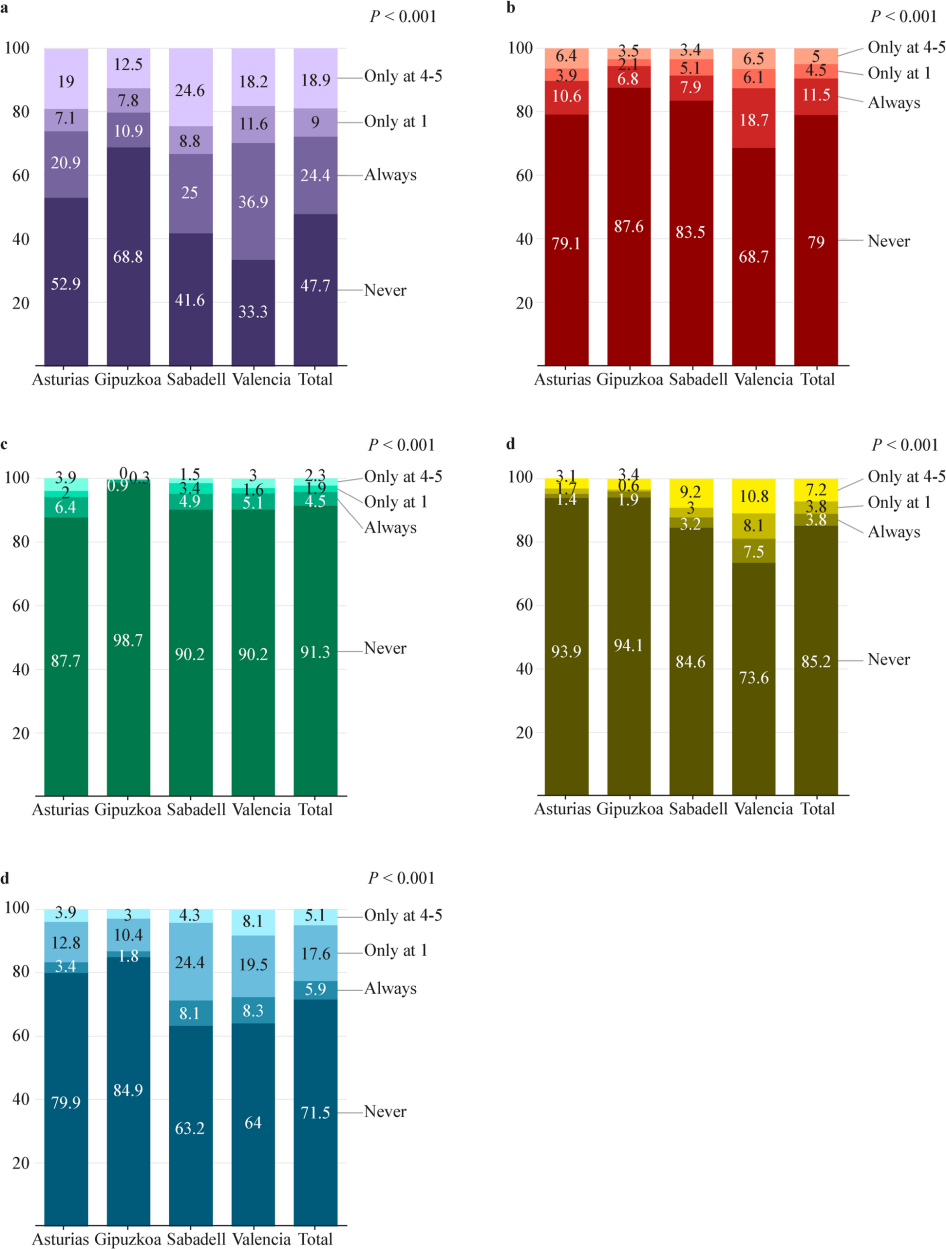
Descriptive analyses for pet ownership.** a** Has the child had any pet? **b** Has the child had any dog? **c** Has the child had any cat? **d** Has the child had any bird? **e** Has the child had any other animals? *P* values from Chi-squared tests for differences between cohorts are all < 0.001

**Fig. 3:**
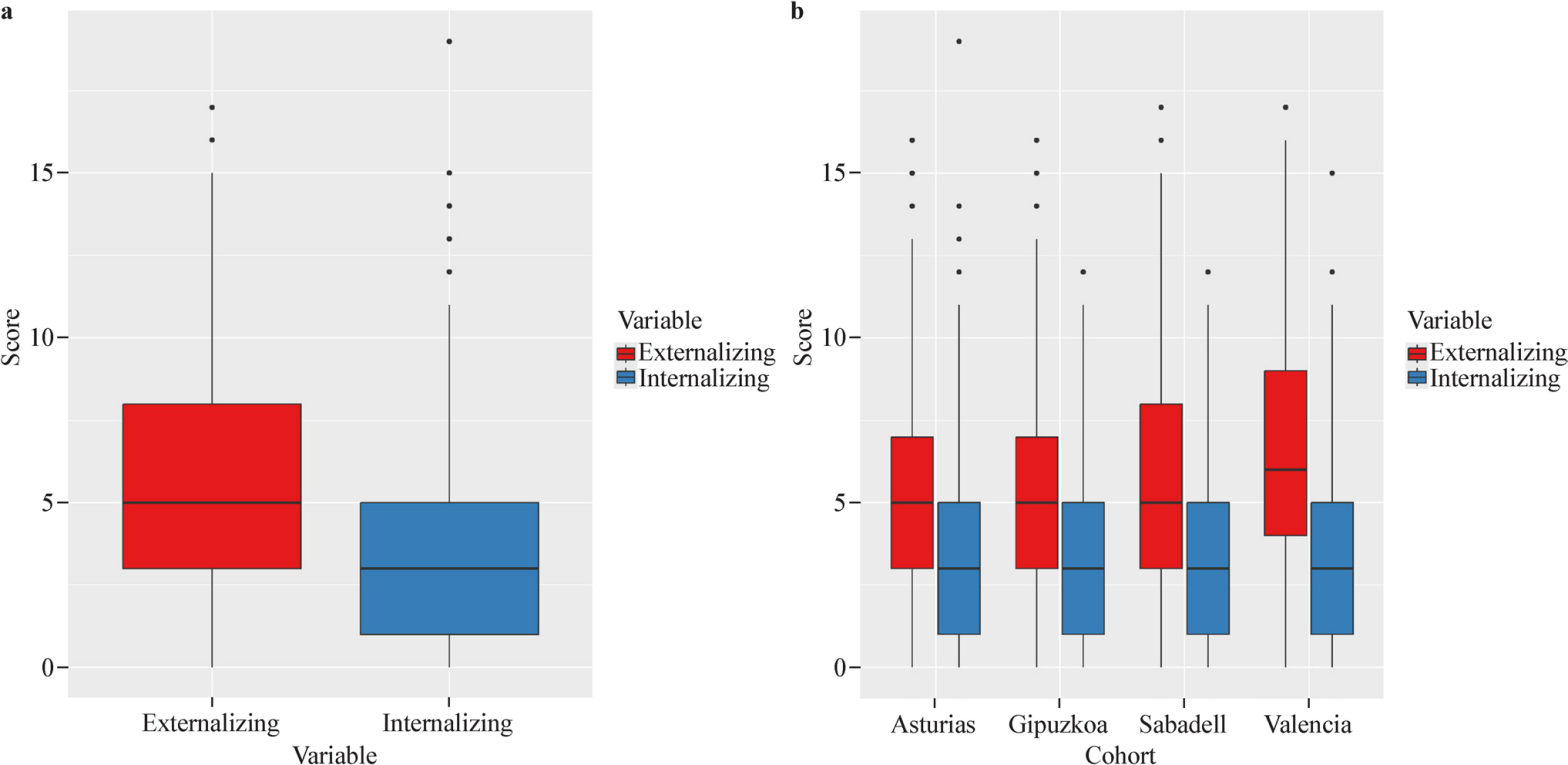
Descriptive analyses for internalizing and externalizing problems. **a** Internalizing and externalizing problems in all cohorts; **b** internalizing and externalizing problems across cohorts

Descriptive analysis of pet ownership is displayed in Fig. 2a–e and *P* values were calculated for differences between cohorts. Briefly, 24.0%, 11.6%, 4.9%, 4.1% and 6.2% of families always owned some kind of pet, dog, cat, bird or other animal, respectively. Cohorts differed in pet ownership for all the animals considered (*P* values < 0.001). In general terms, Valencia was the cohort with more pet owners at both follow-ups (except for cats, which were always more common in Asturias) and Gipuzkoa was the cohort with fewer owners (Supplementary Table 3). Internalizing problems presented a median (Med) of 3 and an interquartile range (IQR) of 1–5, and externalizing problems presented a Med (IQR) of 5 (3–8) (Fig. 3a). Results differed only for externalizing problems 5 (3–7), 5 (3–7), 5 (3–8), and 6 (4–9) for Asturias, Gipuzkoa, Sabadell and Valencia, respectively (*P* < 0.001) (Fig. 3b and Supplementary Tables 4, 5).

### Bivariate analysis

Results of the bivariate analyses are shown in Table 2. In general, the lowest scores were observed for those who never had an animal of any kind. In most cases, having a pet always presented similar but slightly higher scores, whereas, for those who had animals only at one follow-up, the scores tended to be higher. For internalizing problems, this was observed for dogs and especially for cat ownership, with higher scores in the latter category [having a cat only at age 4–5, Med (IQR): 4 (2–6)]. For externalizing problems, this trend was especially true for having any pet, a dog, or other animal. Bivariate analyses disaggregated by cohort are presented in Supplementary Table 4.

**Table 2:** Bivariate analyses: association between pet ownership and internalizing and externalizing problems

Variables	Internalizing				Externalizing			
	Med	P25	P75	*P*^a^	Med	P25	P75	*P*^a^
Any pet								
Never	3	1	5	0.546	5	3	7	0.010
Always	3	1	5		5	3	8	
Only at 1 y	3	2	5		5	3	8	
Only at 4–5 y	3	1	5		6	3	8	
Dog								
Never	3	1	5	0.051	5	3	8	0.026
Always	3	1	5		5	3	8	
Only at 1 y	4	2	6		6	4	9	
Only at 4–5 y	3	1	6		6	4	9	
Cat								
Never	3	1	5	0.049	5	3	8	0.096
Always	3	1	6		5	3	8	
Only at 1 y	2	1	4		5	3	9	
Only at 4–5 y	4	2	6		7	4	9	
Bird								
Never	3	1	5	0.891	5	3	8	0.229
Always	3	2	5		6	3	9	
Only at 1 y	3	1	5		5	3	8	
Only at 4–5 y	3	1	5		6	3	8	
Other animals								
Never	3	1	5	0.197	5	3	8	0.031
Always	2	1	4		5	3	7	
Only at 1 y	3	1	5		6	3	9	
Only at 4–5 y	3	1	5		5	4	8	

*Med* median, *P25* percentile 25, *P75* percentile 75. ^a^*P* values of Kruskal‒Wallis test for differences between groups

Supplementary Table 6a–e present sample characteristics associated with pet ownership. In general, more deprived families had a higher percentage of pet ownership (except for cats). In addition, fathers who smoked and monomarental families (female-headed single-parent families) were positively related to owning any pet, dog or cat. Preterm birth was associated with having any pet or a dog. With the exception of cats, younger mothers also had pets more frequently.

### Multivariate analysis

**Fig. 4:**
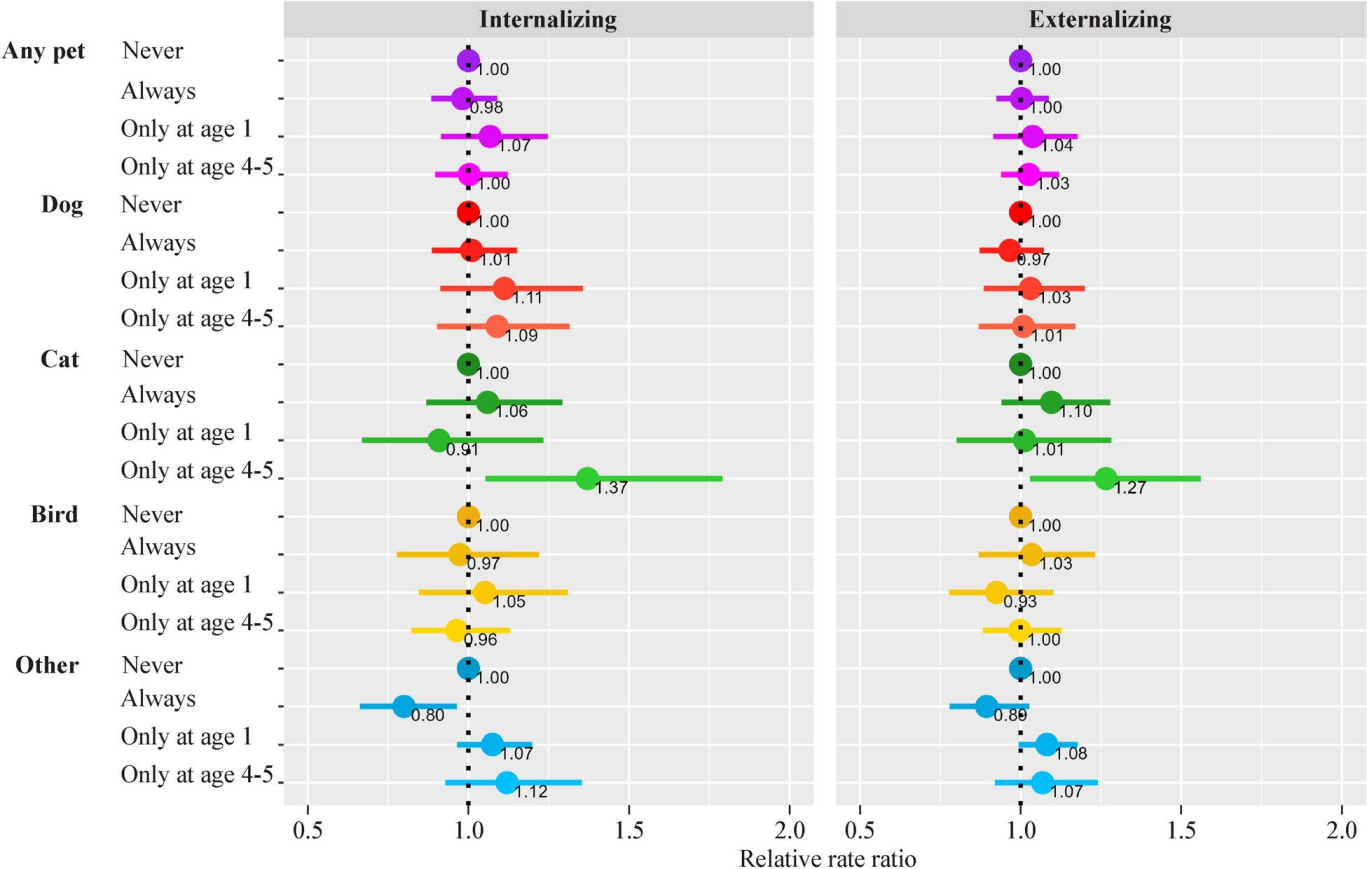
Pet ownership and mental health. Adjusted by age, sex, cohort, maternal education, family social class, and weeks of breastfeeding. In addition, the data were further adjusted for internalizing: maternal country of origin, alcohol intake during pregnancy, smoking, parity, and child's rhinitis; externalizing: paternal country of origin, maternal smoking during pregnancy, and maternal age, paternal smoking (child's age 7–8 years), number of siblings

Results of the adjusted models revealed that owning a cat only at age 4–5 was related to increased risk of internalizing [relative rate ratio (RRR) (95% CI) = 1.37 (1.05–1.79)] and externalizing problems [1.27 (1.03–1.56)]. Owning other animals (different from dogs, cats or birds) at both follow-ups were related to reduced risks for children in terms of internalizing [0.80 (0.66–0.96)] and externalizing [0.89 (0.78–1.03)] problems (Fig. 4). The remainder of the animals did not present significant associations with children’s mental health. In the sensitivity analysis (Supplementary Text), six modifications were tested. First, the IPW procedure was applied (Supplementary Fig. 2). Second, the farm animals were included under the category “other animals” (Supplementary Fig. 3a, b). Third, preterm births were excluded from the analysis (Supplementary Fig. 4). These changes resulted in only minimal variations in the outcome estimates and their statistical significance. Fourth, in the analyses considering the SDQ subscales, similar trends were observed, except for the prosocial scale (Supplementary Fig. 5). Fifth, when we mutually adjusted our exposures, no significant changes were observed (Supplementary Fig. 6). Sixth, in Supplementary Fig. 7, we observe that estimates barely change when ownership and cohabitation was compared at age 4–5, with a slight loss of significance for cat ownership vs cat cohabitation. We explored how cat cohabitation was distributed across our four-category variables for cat ownership. We found that only 32.4% of cohabiting cats were in the “only at 4–5” group, while the majority belonged to the “always” group.

Tukey test and false discovery rates revealed a borderline association between never having cats and having them at 4–5 years with internalizing problems (*P* = 0.063); a link to externalizing problems was ruled out. In addition, having other animals significantly differed from never having them for both internalizing and externalizing problems (*P* = 0.038).

## Discussion

In this work, we explored how the presence of pets at 1 and 4–5 years of age affects child mental health at ages 7–8 years. We aimed to compare the relationship between different types of pets with internalizing and externalizing problems. Pet distribution and externalizing problems varied across the cohorts. We did not find any significant associations between owning any pet, a dog or a bird with internalizing or externalizing problems. A greater risk of internalizing problems was observed with having cats only at the age of 4–5 years and lower risks of internalizing problems were reported when animals other than dogs, cats or birds were present.

We found no positive effects of pets such as dogs, cats or birds on mental health. Whilst some studies suggest pets can improve children's mental health (reduced anxiety, depression and loneliness), self-esteem, behaviour and cognitive, educational and social outcomes [7, 9, 27], the impact of human-animal interaction—especially with dogs—may be influenced by overlooked factors like family structure [8]. In addition, dog ownership has been linked to negative effects on socialization and a potential risk of neuroticism and internalizing problems in adulthood. A recent review also found that up to 13 studies reported no significant mental health benefits from pet ownership.

One study evaluated pet ownership in the Avon Longitudinal Study of Parents and Children cohort. This study revealed an association between dog ownership and higher behavioral problem scores [28], potentially linked to greater exposure to microbes that may impair cognitive function [14]. Cat ownership was also associated with a higher risk of hyperactivity problems, and having a pet occasionally did not differ significantly from always having one [28].

Our study found no positive association between owning dogs, cats or birds and the outcomes we analyzed. Several factors may explain this. We did not account for pet deaths, potentially confounding results for ages 4–5 years [29]. In addition, pet ownership doesn’t always imply cohabitation, as some pets might serve other purposes (e.g., guarding a second home). Unfortunately, data on cohabitation were only available at age 4–5 years and for a subsample of participants (*n* = 1528). Comparative results between ownership and cohabitation are presented in Supplementary Fig. 7. Briefly, comparing ownership and cohabitation at age 4–5 reveals slight differences in the estimates (below 5% of change) and significance, particularly for cats (RRR) (95% CI) = 1.16 (0.99–1.36) and 1.19 (0.95–1.48) for cat ownership and cat cohabitation in internalizing problems, and 1.19 (1.05- 1.34) and 1.15 (0.97–1.37) for cat ownership and cat cohabitation in externalizing problems. However, these differences are unlikely to substantially affect our results. Most studies with positive findings on the impact of pets on mental health focus on middle-aged children, adolescents or young adults; there is a paucity of data on young children. When such analyses are conducted, they often yield results similar to ours [28]. Several factors might explain why exposure to pets in early childhood does not significantly affect mental health in middle childhood. According to Bowlby’s attachment theory, internal working models and attachment bonds are not fully formed until around 18 years of age, suggesting that a bond with an animal might become more beneficial only after that developmental stage [30]. Similarly, self-psychology concepts such as self-esteem and self-concept continue to develop until late adolescence [7]. Caring for an animal during this period might enhance these psychological aspects, as children and adolescents often feel more competent, empathic and kind through such experiences. This could explain the positive effects observed in our analysis for children who always had other pets.

Our study explored the impact of owning pets other than dogs, cats or birds on children’s mental health. These pets, which included small mammals, rodents, fish and reptiles, foster weaker emotional bonds due to their phylogenetic distance from humans [31]. Despite this, consistent ownership of such pets provides protective effects against behavioural and peer-related problems. In a PhD dissertation by Purewal on this topic, children aged 7 years, with "other pets" exhibited higher social anxiety, but by age of 11 years, the same animals positively influenced behavioural and peer issues. This shift may relate to the role these animals play within families. These smaller pets are often regarded as “children’s pets” and are easier to care for. They are frequently used to teach children responsibility and compassion, which may enhance cognitive and behavioural skills, such as planning, memory and impulse control. This developmental process could explain their long-term protective effects [28].

In the present work, we found a consistent pattern for owning a cat at 4–5 years of age and higher scores for internalizing and externalizing problems. However, this finding should be interpreted with caution, as pet ownership does not necessarily imply cohabitation. Nonetheless, we believe this does not significantly compromise our results, since nearly 70% of cohabiting cats were classified under the “always owning a cat” category. Therefore, the potential effect observed in Supplementary Fig. 7 would apply to only about 30% of the cases. Compared with dog owners, cat owners are more neurotic and hyperactive and could affect parent’s assessment of child’s behaviour [28]. Cat ownership is related to negative emotion [4], inattention [32] and decreased well-being [33]. These differences may stem from relational patterns and attachment styles; cats are less affectionate [11], exhibit avoidant attachment [4] and are often chosen by families with children who have mental health conditions, due to their lower maintenance needs [32]. This hypothesis could not be tested in this study, as there was no information on mental health in previous follow-ups. Moreover, different types of pets require different types of interaction: dogs, encourage outdoor activity and interaction, cats are typically indoor pets with limited engagement, potentially contributing to anxiety or depression [33]; other animals are usually kept in cages with restrictions on their movements [28]. Another possible explanation for our findings with cats could be potential toxoplasmosis, which has been associated with higher scores in internalizing and rule-breaking behaviour problems [34]. Infection in adults produces flu-like symptoms, however, in children, it can affect cognitive function and mental health (schizophrenia, depression, mania, or bipolar disorder) [34–37].

This study has limitations. First, sample attrition is common in cohort studies where extensive questionnaires, tests and long follow-ups may cause participant fatigue. The INMA project addressed this with annual feedback, warm personal attention and IPW techniques to reduce selection bias. Second, we did not measure pet attachment rather pet ownership, this difference, could have shed some light on our results. Third, we did not describe all combinations of types of pets. An analysis considering these combinations separately and taking into account the wide variety of pets would have been impractical. However, in our last sensitivity analysis, to control for other pets, we mutually adjusted exposure. Finally, there was no information on pet ownership when children were aged 2–3 years, as no in-person follow-ups were conducted during this period. This intermediate exposure could have offered valuable insights into potential trends in mental health.

This study has strengths. First, it compared different follow-ups to assess the impact of pet ownership on child mental health using prospective cohort data, enabling analysis of middle-term exposures and outcomes. Second, the large sample size improved statistical power. Third, exposure was measured before outcomes, avoiding reverse causation. Fourth, advanced methods such as false discovery rate tests and IPW enhanced robustness. Fifth, adjustments for confounders used variables from multiple time points. Sixth, the SDQ [20] ensured valid, comparable mental health assessments. Finally, this is the first study examining the impact of different types of pet ownership during preschool on mental health at primary school entry.

In conclusion, our data showed no significant association for most pets. However, cat ownership at 4–5 years was linked to negative effects on internalizing and externalizing problems, while consistent ownership of other pets appeared beneficial in preventing these issues. Collectively, our data highlights the complex relationship between early pet ownership and child mental health.

## Supplementary Information

Below is the link to the electronic supplementary material.

Supplementary file1 (PDF 3460 KB)

## Data Availability

The datasets generated and/or analysed during the current study are not publicly available due to preservation of participants’ privacy, but are available from the corresponding author on reasonable request and after INMA’s Executive Committee approval.
