# Supplementary material for: Impact of pet ownership in early childhood at ages 1 and 4–5 years on mental health at ages 7–8: findings from the INMA project
Source: World J Pediatr. 2025 Oct 4;21(10):1003–16. doi: 10.1007/s12519-025-00942-2 (PMC12578733; doi:10.1007/s12519-025-00942-2)
Supplement: Supplementary file 1 — Supplementary file1 (PDF 3460 KB) [file 12519_2025_942_MOESM1_ESM.pdf]

Supplementary materials

Supplementary Text

Five sensitivity analyses were performed. The first was to control for sample attrition as significant differences were observed between participants and non-participants at the 7–8-year follow-up. To do so, we used the inverse probability weighting method [1]. Weights were calculated considering those covariates associated with participants' attendance at the 7-8-year follow-up. In particular, we fitted a logistic regression model with attendance at follow-up as the dependent variable and family social class, maternal education, parental country of origin, parity, maternal smoking, age during pregnancy and cohort as predictors of participation and derived propensity-stabilized weights. We then performed weighted regressions to derive the association estimates of interest. The second, included farm animals in the "other animals" exposure variable; and the third excluded preterm children. The fourth analysis, disaggregating by subscale, was also performed to check the robustness of our findings in the broadband scales (internalizing and externalizing problems). In the fifth and last of our sensitivity analyses, we adjusted each model for the remaining exposures to control for the residual effect of the ownership of other pets.

1. Carry PM, Vanderlinden LA, Dong F, Buckner T, Litkowski E, Vigers T, et al. Inverse probability weighting is an effective method to address selection bias during the analysis of high dimensional data. Genet Epidemiol. 2021;45:593–603.

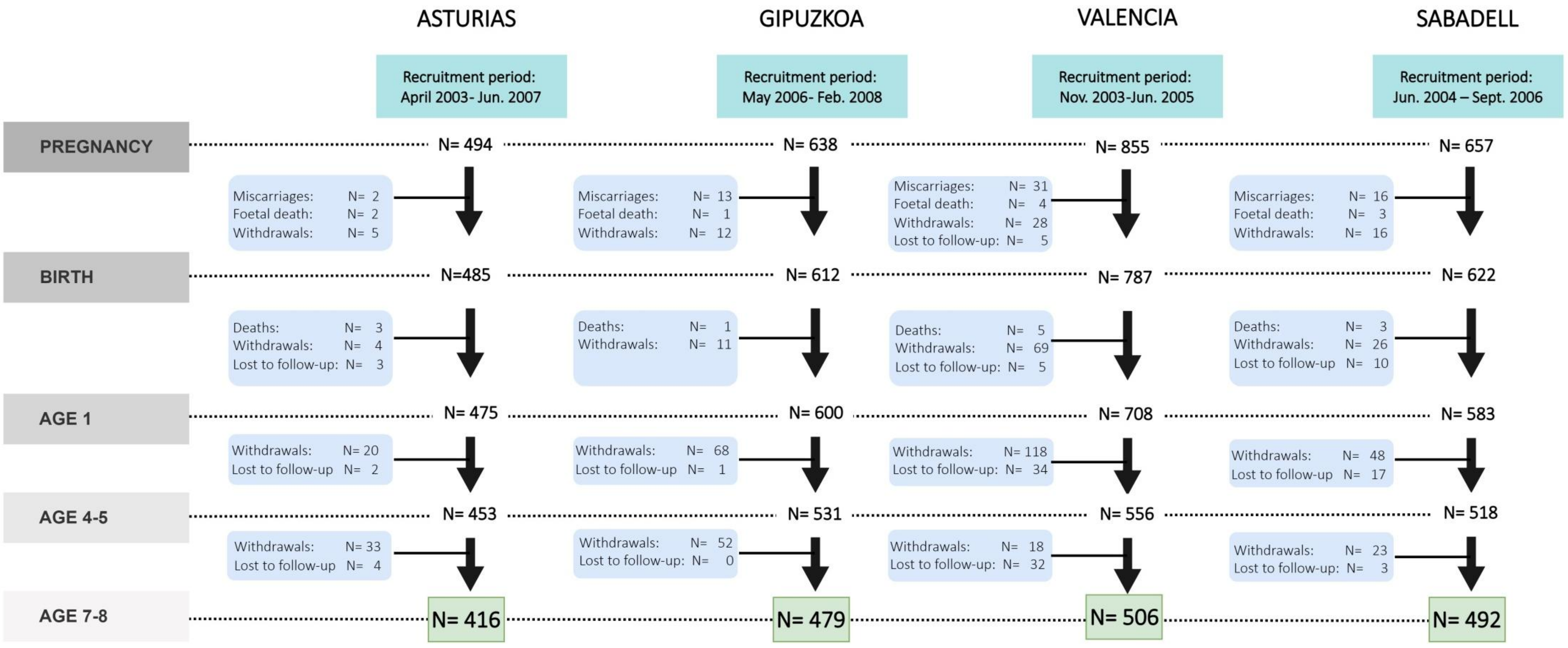

Supplementary Fig. 1 Follow-up visits and sample evolution in each cohort

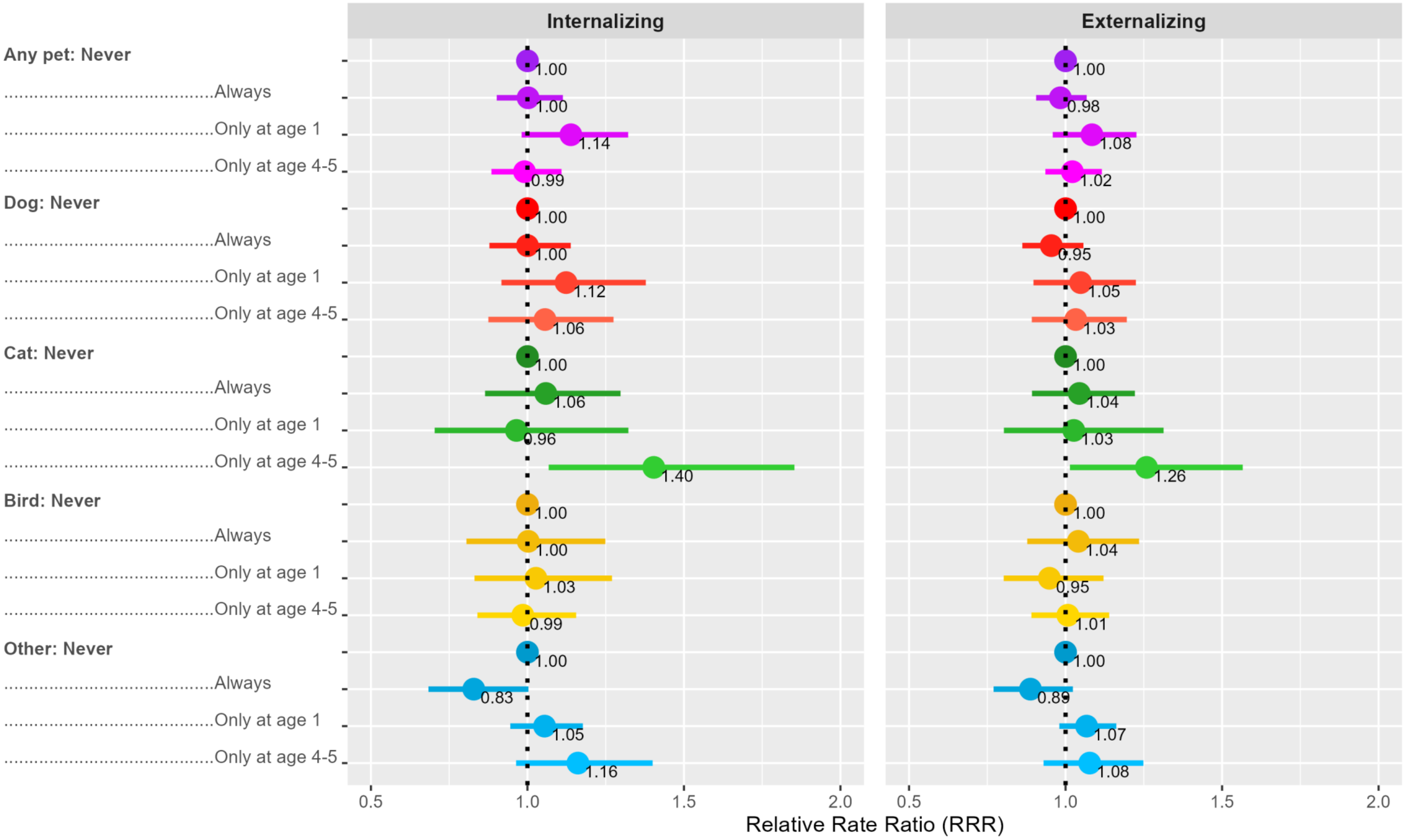

Supplementary Fig. 2 Pet ownership and mental health (inverse probability weighting). Adjusted by age, sex, cohort, maternal education, family social class, and weeks of breastfeeding. Additionally adjusted for: internalizing: maternal country of origin, alcohol intake during pregnancy, smoking, parity, and child's rhinitis; externalizing: maternal smoking during pregnancy, and maternal age, paternal smoking (child's age 7-8), small for gestational age

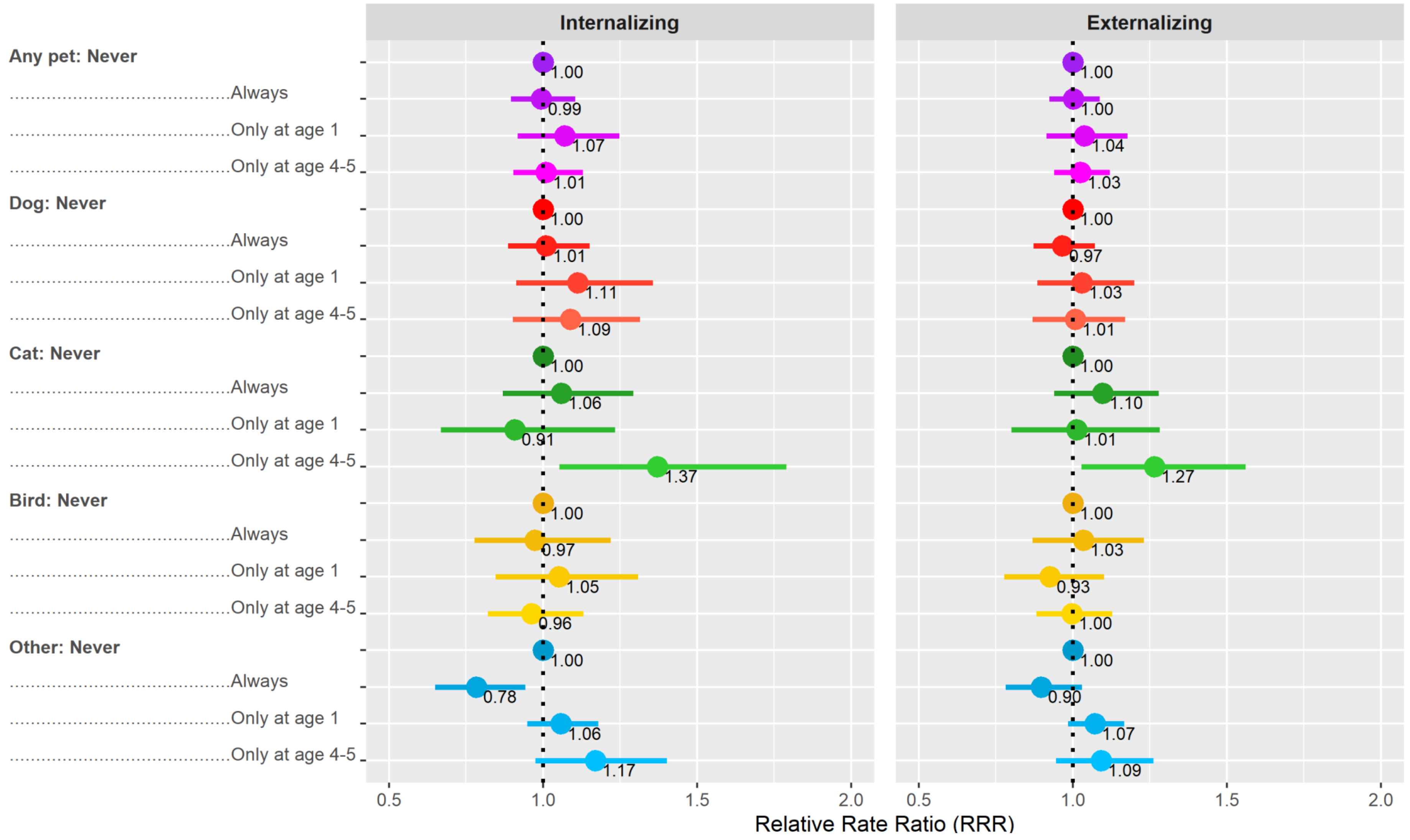

Supplementary Fig. 3a Pet ownership and mental health with farm animals. Adjusted by age, sex, cohort, maternal education, family social class, and weeks of breastfeeding. Additionally adjusted for: internalizing: maternal country of origin, alcohol intake during pregnancy, smoking, parity, and child's rhinitis; externalizing: paternal country of origin, maternal smoking during pregnancy, and maternal age, paternal smoking (child's age 7-8), number of siblings

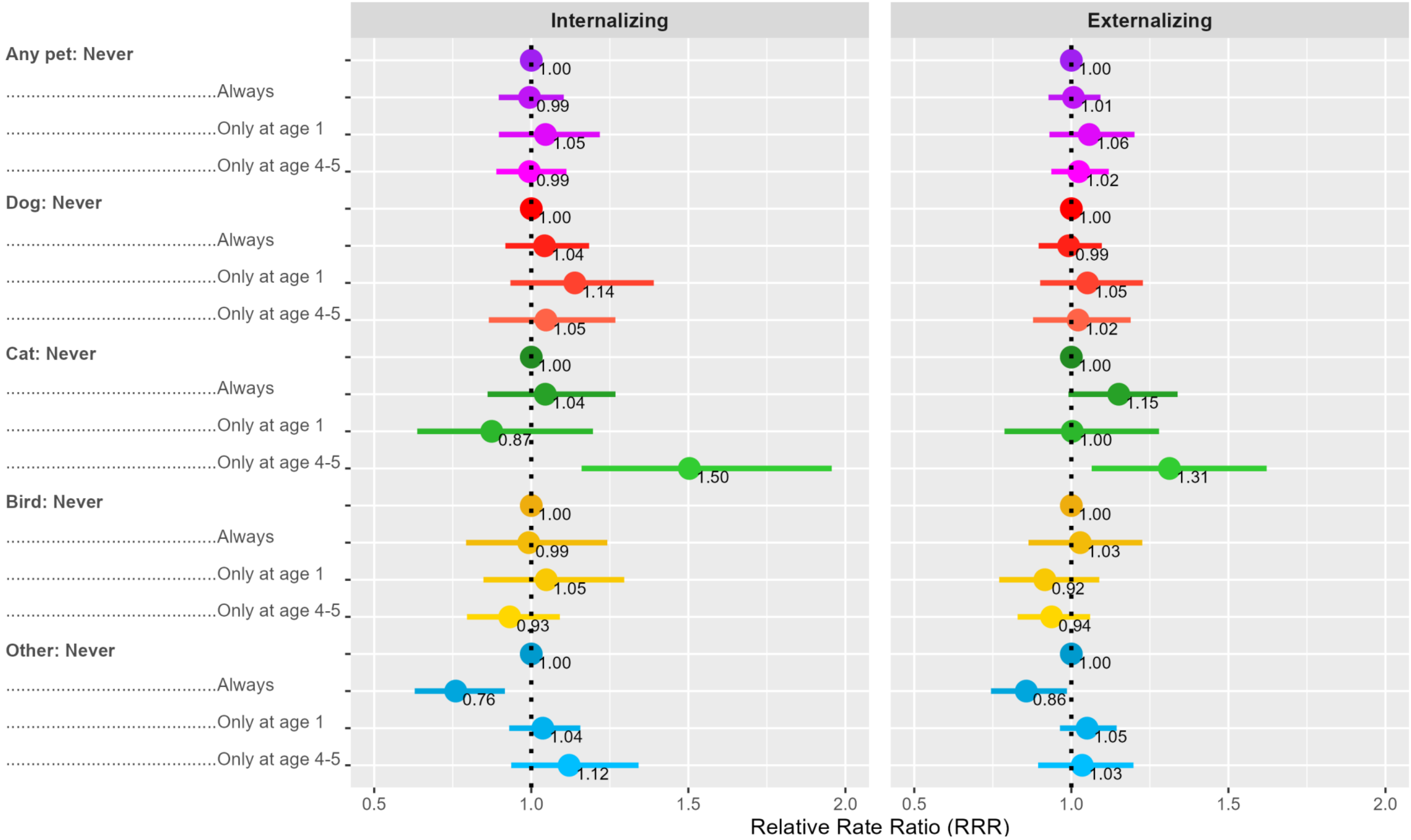

Supplementary Fig. 3b Pet ownership and mental health with farm animals (inverse probability weighting). Adjusted by age, sex, cohort, maternal education, family social class, and weeks of breastfeeding. Additionally adjusted for: internalizing: maternal country of origin, alcohol intake during pregnancy, smoking, parity, and child's rhinitis; externalizing: maternal smoking during pregnancy, and maternal age, paternal smoking (child's age 7-8), small for gestational age

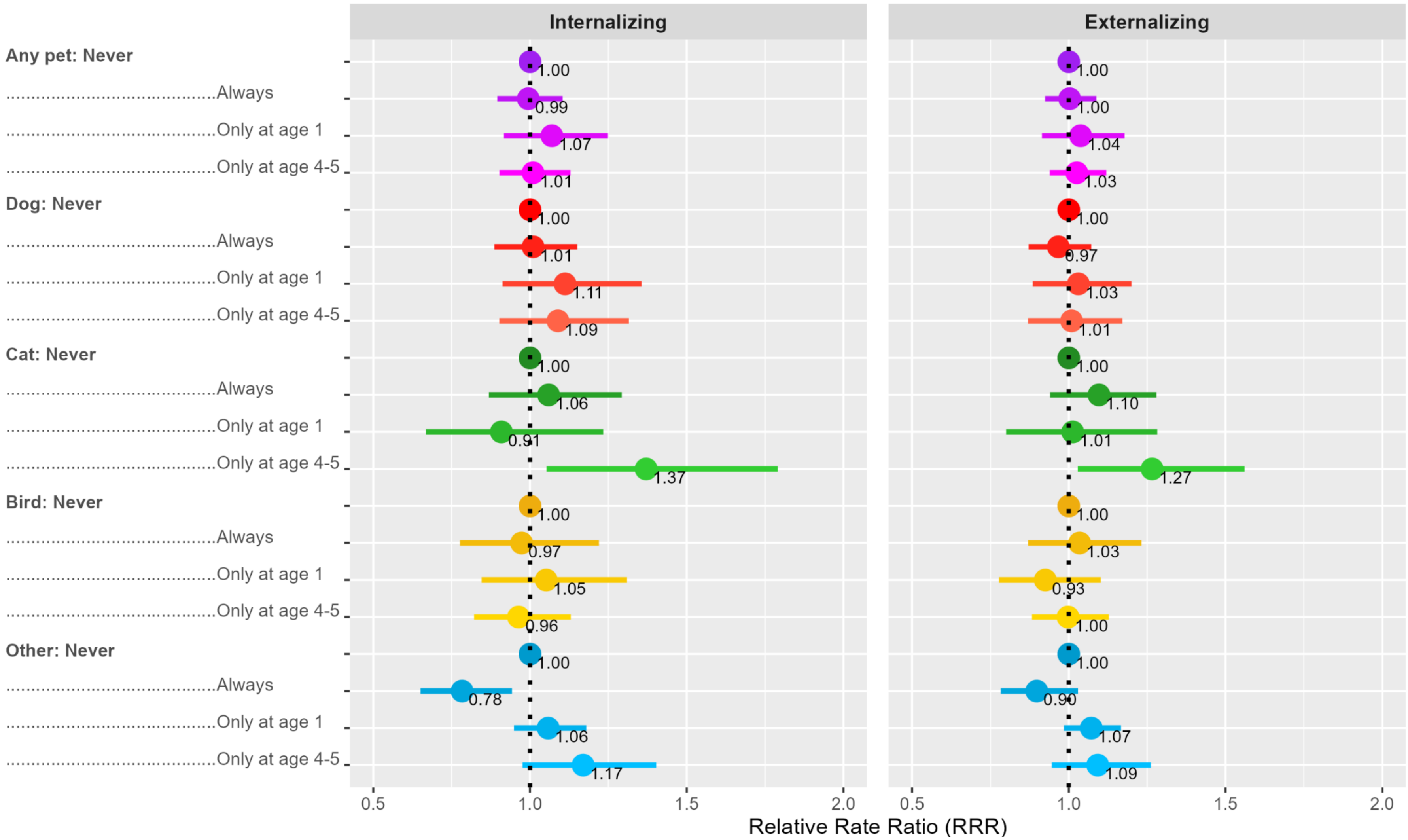

Supplementary Fig. 4a Pet ownership and mental health with farm animals excluding preterm children. Adjusted by age, sex, cohort, maternal education, family social class, and weeks of breastfeeding. Additionally adjusted for: internalizing: maternal country of origin, alcohol intake during pregnancy, smoking, parity, and child's rhinitis; externalizing: maternal smoking during pregnancy, and maternal age, paternal smoking (child's age 7-8), small for gestational age

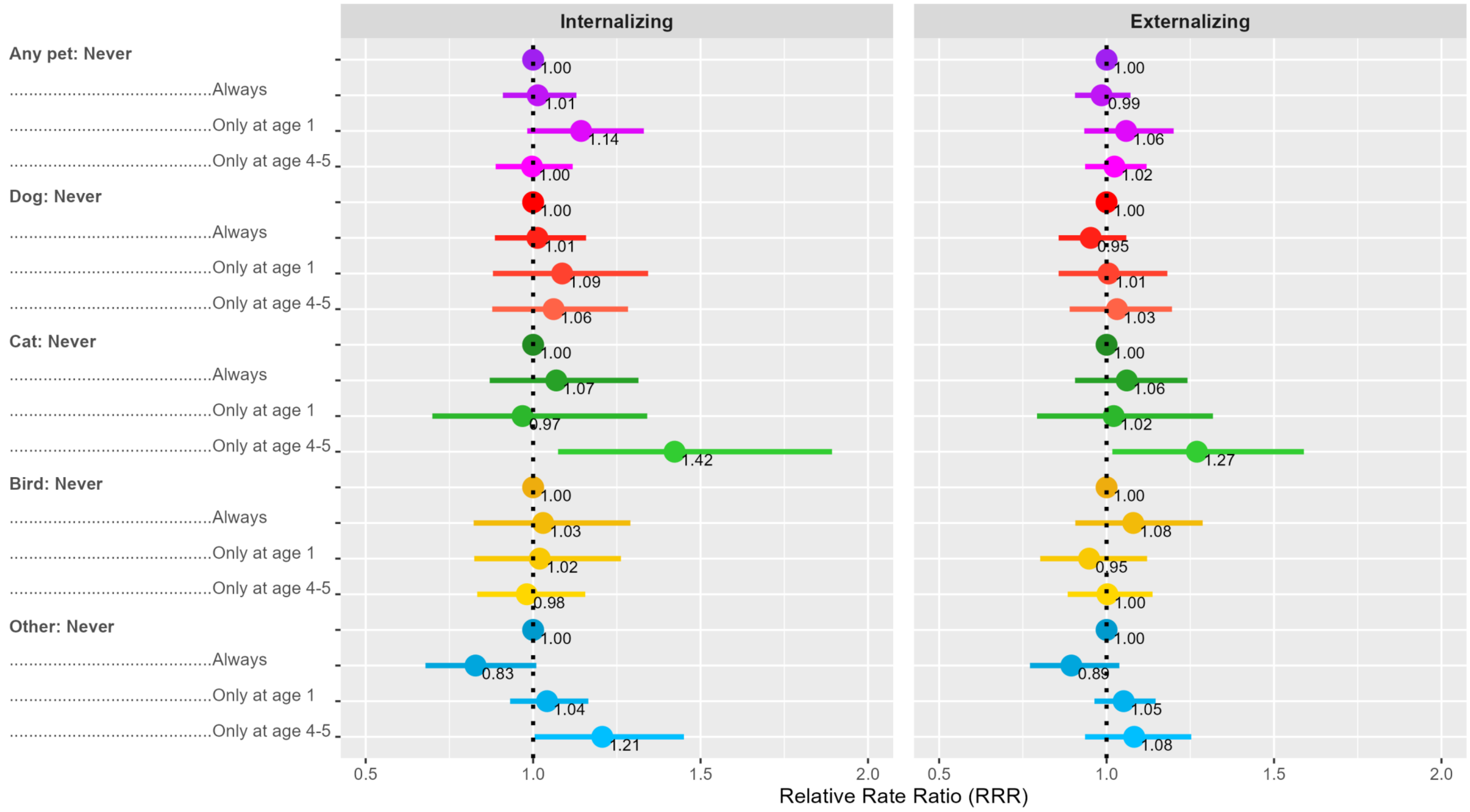

Supplementary Fig. 4b Pet ownership and mental health with farm animals excluding preterm children (inverse probability weighting). Adjusted by age, sex, cohort, maternal education, family social class, and weeks of breastfeeding. Additionally adjusted for: internalizing: maternal country of origin, alcohol intake during pregnancy, smoking, parity, and child's rhinitis; externalizing: maternal smoking during pregnancy, and maternal age, paternal smoking (child's age 7-8), small for gestational age

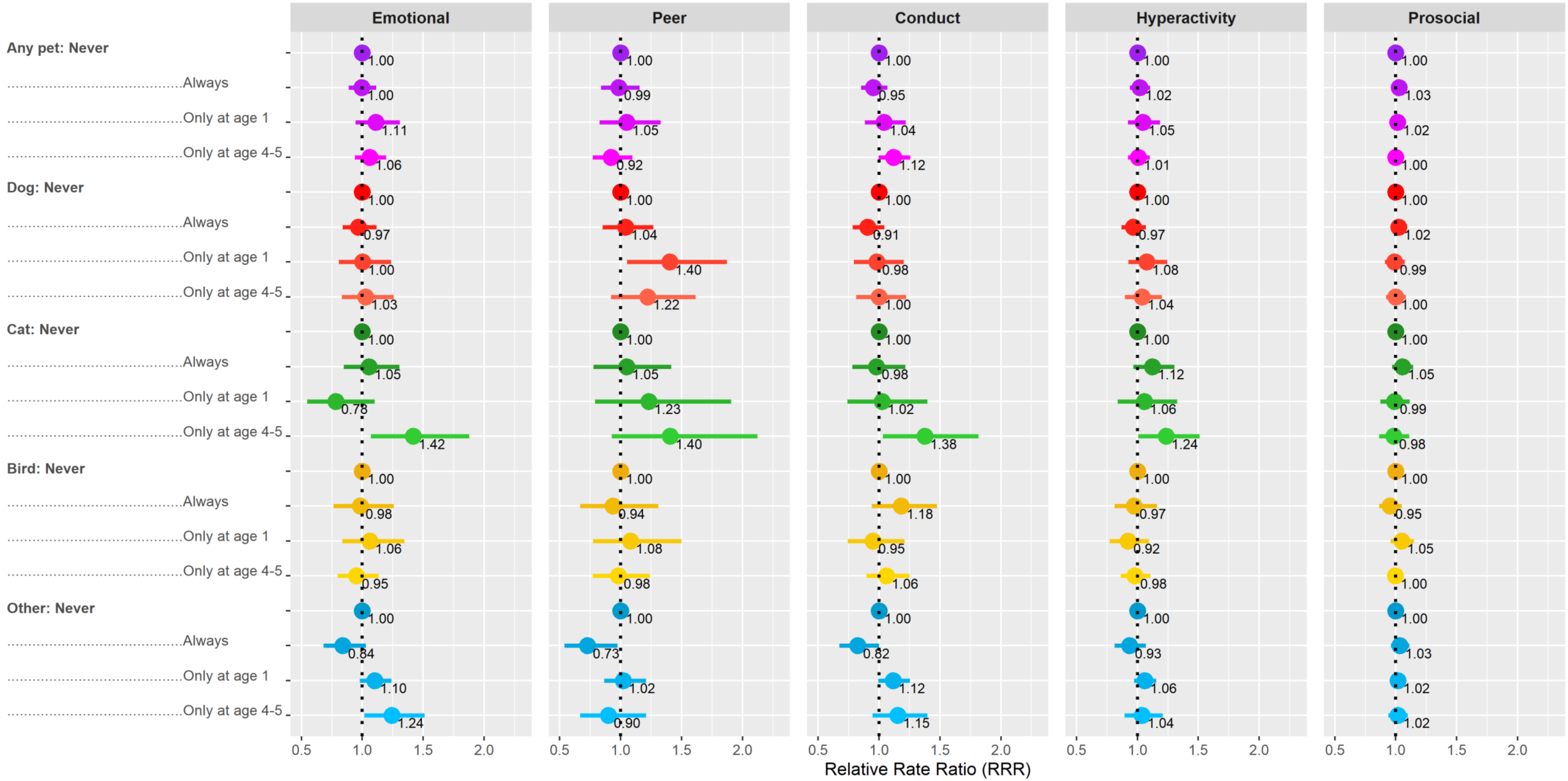

Supplementary Fig. 5 Pet ownership and Strengths and Difficulties Questionnaire (SDQ) subscales. Adjusted by age, sex, and cohort. Additionally adjusted for: emotional: maternal education, parity, weeks of breastfeeding, maternal smoking (7-8), type of zone (birth), rhinitis symptoms; peer: maternal education, parity, weeks of breastfeeding, maternal country of origin, alcohol , family type (7-8), paternal age, rhinitis diagnose; conduct: maternal education, family social class, maternal smoking (7-8), paternal smoking (7-8), family type (7-8); hyperactivity: maternal and paternal education, weeks of breastfeeding, paternal smoking (7-8), maternal age, maternal smoking , number of siblings (4-5); prosocial: child's physical activity at age 7-8

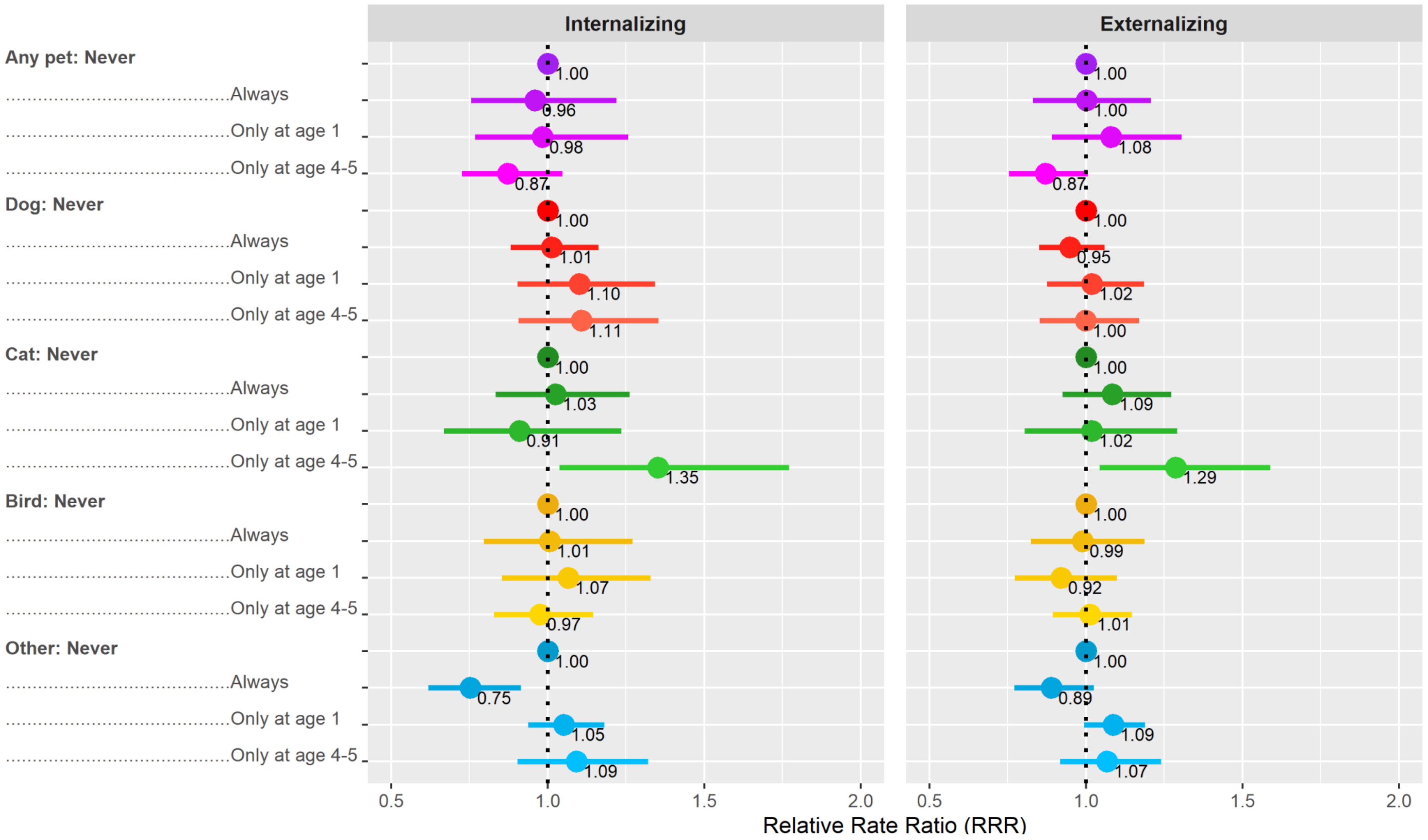

Supplementary Fig. 6 Pet ownership and mental health mutually adjusted. Adjusted by age, sex, cohort, maternal education, family social class, and weeks of breastfeeding. Additionally adjusted for: internalizing: maternal country of origin, alcohol intake during pregnancy, smoking, parity, and child's rhinitis; externalizing: maternal smoking during pregnancy, and maternal age, paternal smoking (child's age 7-8), small for gestational age. Each model was additionally adjusted by the other exposures

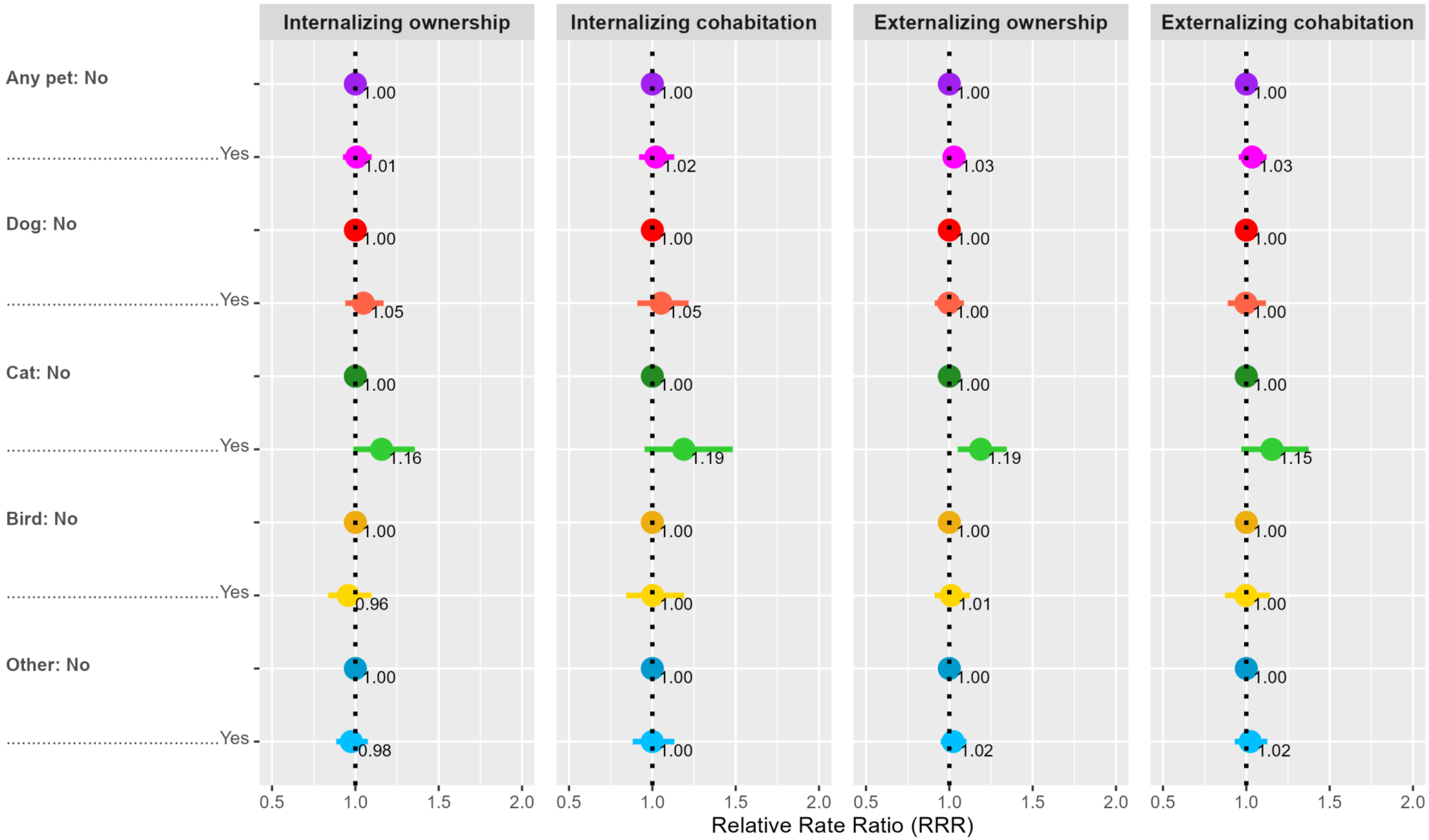

Supplementary Fig. 7 Pet ownership vs pet cohabitation and mental health at age 4-5. Adjusted by age, sex, cohort, maternal education, family social class, and weeks of breastfeeding. Additionally adjusted for: internalizing: maternal country of origin, alcohol intake during pregnancy, smoking, parity, and child's rhinitis; externalizing: maternal smoking during pregnancy, and maternal age, paternal smoking (child's age 7-8), small for gestational age, *n* = 1528

Supplementary Table 1. Differences between non-participants and participants at 7-8 years of age

Variables		Non-included		Included		
		n	%	*n*	%	*P*
Family social class	Higher (I + II)	192	21.4	619	36.2	< 0.001
	Middle (III)	204	22.7	441	25.8	
	Lower (IV + V)	502	55.9	648	37.9	
Maternal education level	Up to primary	322	35.7	341	19.4	< 0.001
	Secondary	367	40.7	740	42.1	
	University	213	23.6	678	38.5	
Paternal education level	Up to primary	406	43.8	565	32.0	< 0.001
	Secondary	383	41.3	777	44.0	
	University	138	14.9	424	24.0	
Maternal country of origin	Spain	799	84.2	1667	94.3	< 0.001
	Not Spain	150	15.8	101	5.7	
Paternal country of origin	Spain	802	84.7	1656	93.5	< 0.001
	Not Spain	145	15.3	116	6.5	
Parity	0	478	53.3	986	57.8	< 0.001
	1	330	36.8	628	36.8	
	2+	88	9.8	93	5.4	
Maternal smoking during pregnancy	No	634	76.9	1451	83.6	< 0.001
	Yes	190	23.1	285	16.4	
Paternal smoking during pregnancy	No	659	79.8	1486	85.5	< 0.001
	Yes	167	20.2	252	14.5	
Maternal alcohol during pregnancy	No	728	89.1	1567	90.9	0.155
	Yes	89	10.9	157	9.1	
Paternal alcohol during pregnancy	No	234	26.2	389	23.1	0.080
	Yes	660	73.8	1297	76.9	
Type of zone (birth)	Urban	795	92.9	1607	94.1	0.234
	Rural	61	7.1	101	5.9	
Anxiety history	No	141	14.8	241	13.6	0.373
	Yes	811	85.2	1535	86.4	
Depression history	No	104	10.9	163	9.2	0.143
	Yes	848	89.1	1613	90.8	
Preterm	No	790	94.5	1693	96.0	0.077
	Yes	46	5.5	70	4.0	
Small for gestational age for weight (INMA table) (birth)	No	699	90.3	1515	90.0	0.051
	Yes	75	9.7	168	10.0	
Small for gestational age for head circumference (INMA table) (birth)	No	672	89.0	1480	90.2	0.790
	Yes	83	11.0	161	9.8	
Sex of the child	Female	402	47.7	866	48.7	0.626
	Male	441	52.3	912	51.3	
Cohort	Asturias	132	13.4	362	20.4	< 0.001
	Gipuzkoa	241	24.4	397	22.3	
	Sabadell	228	23.1	549	30.9	
	Valencia	385	39.0	470	26.4	

	Non-included			Included			
Continuous variables	Med	P25	P75	Med	P25	P75	*P*
Maternal age	30	26.8	33	31	28.5	34	< 0.001
Paternal age	32	29	36	33	30	36	< 0.001

Med median, *P25* percentile 25, *P75* percentile 75

Supplementary Table 2. Differences among cohorts for sample characteristics

Variables		Asturias		Gipuzkoa		Sabadell		Valencia		
		n	%	*n*	%	*n*	%	*n*	%	*P*[a]
Sociodemographic characteristics										
Family social class	Higher (I + II)	137	33.0	226	47.2	167	33.9	144	28.5	< 0.001
	Middle (III)	94	22.7	104	21.7	147	29.9	136	26.9	
	Lower (IV + V)	184	44.3	149	31.1	178	36.2	226	44.7	
Maternal education level	Up to primary	64	15.4	55	11.5	120	24.5	138	27.7	< 0.001
	Secondary	186	44.7	173	36.3	212	43.4	221	43.7	
	University	166	39.9	249	52.2	157	32.1	147	29.1	
Paternal education level	Up to primary	126	30.4	102	21.5	164	33.6	216	42.9	< 0.001
	Secondary	191	46.0	233	49.1	211	43.2	199	39.5	
	University	98	23.6	140	29.5	113	23.2	89	17.7	
Maternal employment (4-5 y)	Working	259	67.6	296	77.1	345	75.5	361	71.9	0.003
	Not working	61	15.9	51	13.3	67	14.7	81	16.1	
	Homemaker	63	16.4	37	9.6	45	9.8	60	12.0	
Paternal employment (4-5 y)	Working	347	92.5	363	95.0	410	91.5	428	86.6	< 0.001
	Not working	28	7.5	19	5.0	38	8.5	66	13.4	
Maternal country of origin	Spain	403	96.9	466	97.3	448	91.6	470	93.3	< 0.001
	Not Spain	13	3.1	13	2.7	41	8.4	34	6.7	
Paternal country of origin	Spain	404	97.1	470	98.1	443	90.2	449	89.1	< 0.001
	Not Spain	12	2.9	9	1.9	48	9.8	55	10.9	
Parental use of tobacco and alcohol										
Maternal smoking during pregnancy	No	324	82.7	410	87.8	420	86.6	394	77.9	< 0.001
	Yes	68	17.3	57	12.2	65	13.4	112	22.1	
Paternal smoking during pregnancy	No	328	83.5	417	89.1	425	87.4	403	79.6	< 0.001
	Yes	65	16.5	51	10.9	61	12.6	103	20.4	
Maternal smoking (7-8 y)	No	283	79.1	325	82.1	342	72.3	316	67.4	< 0.001
	Yes	75	20.9	71	17.9	131	27.7	153	32.6	
Paternal smoking (7-8 y)	No	260	72.8	301	76.6	309	66.7	289	63.9	< 0.001
	Yes	97	27.2	92	23.4	154	33.3	163	36.1	
Maternal alcohol during pregnancy	No	354	91.1	433	93.7	432	89.1	449	89.3	< 0.001
	Yes	31	8.1	29	6.3	53	10.9	54	10.7	
Paternal alcohol during pregnancy	No	141	34.1	85	18.3	86	17.6	128	25.4	< 0.001
	Yes	272	65.9	379	81.7	403	82.4	375	74.6	
Family characteristics and organization										
Parity	0	260	62.5	270	56.4	282	57.6	282	55.7	0.079
	1	135	32.5	183	38.2	180	36.7	191	37.7	
	2+	21	5.0	26	5.4	28	5.7	33	6.5	
Number of siblings (4-5 y)	Only child	162	43.3	69	18.0	136	28.4	149	29.6	< 0.001
	1 sibling	190	50.8	269	70.2	302	63.0	302	60.0	
	2 or more	22	5.9	45	11.7	41	8.6	52	10.3	

Variables		Asturias		Gipuzkoa		Sabadell		Valencia		
		n	%	*n*	%	*n*	%	*n*	%	P^a
Family characteristics and organization										
Mother living with... (7-8 y)	Father	316	90.0	368	93.2	408	87.2	405	86.5	< 0.001
	Other partner	12	3.4	14	3.5	41	8.8	27	5.8	
	No partner	23	6.6	13	3.3	19	4.1	26	5.6	
	Grandparents and other	0	0.0	0	0.0	0	0.0	10	2.1	
Nursery before 24 mon of age	No	223	56.5	133	33.6	196	43.8	137	27.3	< 0.001
	Yes	172	43.5	263	66.4	252	56.3	365	72.7	
Both parents are the main carer (4-5 y)	No	275	71.8	264	68.6	304	62.8	381	75.7	< 0.001
	Yes	108	28.2	121	31.4	180	37.2	122	24.3	
	House	59	16.0	18	5.0	13	3.3	61	12.5	< 0.001
	Terraced	20	5.4	32	8.8	56	14.3	106	21.7	
	Flat	288	78.3	313	86.2	320	81.8	319	65.2	
Type of dwelling (4-5 y)	Other	1	0.3	0	0.0	2	0.5	3	0.6	
Type of zone (4-5 y)	No rural	341	86.6	441	95.0	490	100.0	434	92.7	< 0.001
	Rural	52	13.2	23	5.0	0	0.0	34	7.3	
Clinical factors										
Small for gestational age for weight (INMA table) (birth)	No	370	92.0	432	91.9	432	89.1	446	88.3	0.074
	Yes	32	8.0	38	8.1	53	10.9	59	11.7	
Small for gestational age for head circumference (INMA table) (birth)	No	355	90.3	395	89.4	425	89.7	457	90.7	0.998
	Yes	38	9.7	47	10.6	49	10.3	47	9.3	
Preterm	No	385	94.1	461	96.6	473	97.5	478	94.7	< 0.001
	Yes	24	5.9	16	3.4	12	2.5	27	5.3	
Child's rhinitis (blocked nose)	No	247	69.2	297	75.2	402	84.8	368	78.6	< 0.001
	Yes	110	30.8	98	24.8	72	15.2	100	21.4	
Diagnosed with rhinitis	No	340	95.0	366	100.0	464	98.1	451	96.4	< 0.001
	Yes	18	5.0	0	0.0	9	1.9	17	3.6	
Sex of the child	Female	194	46.6	244	50.9	238	48.4	244	48.2	0.717
	Male	222	53.4	235	49.1	254	51.6	262	51.8	

	Asturias			Gipuzkoa			Sabadell			Valencia			
Continuous variables	Med	P25	P75	Med	P25	P75	Med	P25	P75	Med	P25	P75	P^b
Maternal age	31.0	29.0	35.0	31.0	29.0	34.0	30.0	28.0	33.0	30.0	28.0	33.0	< 0.001
Paternal age	34.0	30.0	37.0	33.0	31.0	6.0	32.0	29.0	35.0	32.0	29.0	35.0	< 0.001
Child's age	8.3	8.1	8.4	7.7	7.7	7.8	6.8	6.5	7.5	7.5	7.4	7.6	< 0.001
Apgar 1 min (birth)	9.0	9.0	9.0	9.0	9.0	10.0	9.0	9.0	9.0	9.0	9.0	10.0	< 0.001
Weeks of breastfeeding	13.1	0.0	26.0	26.1	13.0	43.7	12.9	12.9	21.6	21.6	4.3	39.3	< 0.001
Maternal intelligence (WAIS-III) (4-5 y)	98.8	91.5	106.1	98.8	87.8	109.9	91.5	91.5	98.8	98.8	91.5	109.8	< 0.001
Total extracurricular physical activity (h/d)	1.0	0.0	1.0	1.0	0.0	2.0	1.0	1.0	1.0	1.0	1.0	2.0	< 0.001

INMA Infancia y Medio Ambiente, *Med* median, *P25* percentile 25, *P75* percentile 75. [a]*P* values from Chi-squared tests for differences among cohorts; [b]*P* values from Kruskal–Wallis tests for differences among cohorts

Supplementary Table 3. Frequencies and Chi-square tests of pet ownership across cohorts

Variables		Asturias		Gipuzkoa		Sabadell		Valencia		
		n	%	*n*	%	*n*	%	*n*	%	*P*[a]
Has the child had any pet?	Never	200	52.9	265	68.8	198	41.6	166	33.3	< 0.001
	Always	79	20.9	42	10.9	119	25.0	184	36.9	
	Only at 1	27	7.1	30	7.8	42	8.8	58	11.6	
	Only at 4-5	72	19.0	48	12.5	117	24.6	91	18.2	
Presence of dog	Never	283	79.1	298	87.6	390	83.5	338	68.7	< 0.001
	Always	38	10.6	23	6.8	37	7.9	92	18.7	
	Only at 1	14	3.9	7	2.1	24	5.1	30	6.1	
	Only at 4-5	23	6.4	12	3.5	16	3.4	32	6.5	
Presence of cat	Never	314	87.7	313	98.7	422	90.2	444	90.2	< 0.001
	Always	23	6.4	3	0.9	23	4.9	25	5.1	
	Only at 1	7	2.0	0	0.0	16	3.4	8	1.6	
	Only at 4-5	14	3.9	1	0.3	7	1.5	15	3.0	
Presence of bird	Never	336	93.9	302	94.1	395	84.6	362	73.6	< 0.001
	Always	5	1.4	6	1.9	15	3.2	37	7.5	
	Only at 1	6	1.7	2	0.6	14	3.0	40	8.1	
	Only at 4-5	11	3.1	11	3.4	43	9.2	53	10.8	
Presence of other animals	Never	286	79.9	286	84.9	296	63.2	315	64.0	< 0.001
	Always	12	3.4	6	1.8	38	8.1	41	8.3	
	Only at 1	46	12.8	35	10.4	114	24.4	96	19.5	
	Only at 4-5	14	3.9	10	3	20	4.3	40	8.1	

[a]*P* values of Chi-square tests for differences among cohorts

Supplementary Table 4. Internalizing and externalizing problems across cohorts

	Internalizing				Externalizing			
Variables	Med	P25	P75	*P*[b]	Med	P25	P75	*P*[a]
Asturias	3.0	1.0	5.0		5.0	3.0	7.0	
Gipuzkoa	3.0	1.0	5.0		5.0	3.0	7.0	
Sabadell	3.0	1.0	5.0		5.0	3.0	8.0	
Valencia	3.0	1.0	5.0	0.733	6.0	4.0	9.0	< 0.001
Total	3.0	1.0	5.0		5.0	3.0	8.0	

Med median, *P25* percentile 25, *P75* percentile 75. [a]*P* values of Kruskal–Wallis test for differences between groups

Supplementary Table 5. Pet ownership association with internalizing and externalizing problems disaggregated by cohort

Variables		ASTURIAS								GIPUZKOA								SABADELL								VALENCIA							
		Internalizing				Externalizing				Internalizing				Externalizing				Internalizing				Externalizing				Internalizing				Externalizing			
		Med	P25	P75	P^a	Med	P25	P75	P^a	Med	P25	P75	P^a	Med	P25	P75	P^a	Med	P25	P75	P^a	Med	P25	P75	P^a	Med	P25	P75	P^a	Med	P25	P75	P^a
Has the child had any pet?	Never	3	1	5	0.214	5	3	8	0.815	3	1	5	0.403	5	3	7	0.310	3	1	5	0.652	5	2	7	0.211	3	1	5	0.964	6	4	8	0.378
	Always	2	1	4		5	3	7		3	1	6		5	3.5	8		3	1	5		5	2	8		3	1	5		6	3	9	
	Only at 1	4	2	7		5.5	2.5	8		3	2	5		5	3	6		3	2	6		5	3	9		3	1	4		6	5	9	
	Only at 4-5	3	1	4		5	3	7		2	1	4		6	3	8		3	1	5		5.5	3	9		3	1	4		6	4	9	
Presence of dog	Never	2	1	4	0.115	5	2	7	0.449	3	1	5	0.633	5	3	7	0.595	3	1	5	0.132	5	3	7	0.712	3	1	5	0.854	6	4	8	0.011
	Always	3	1	6.5		6	4	8.5		3	1	6		5	3	8		4	1	5		5	2	9		3	1	4		5	3	8	
	Only at 1	5	2	7.5		6.5	2.5	10		3	2	8		5	2	6		4	1	6		6	4	9		3	2	5		6	5	10	
	Only at 4-5	3	2	6		4	3	7		2	1	4		6	4	8		4	1	6		5	2	8		3.5	1	5		7	5.5	10.5	
Presence of cat	Never	3	1	5	0.076	5	3	8	0.134	3	1	5	0.020	5	3	7	0.313	3	1	5	0.879	5	2	8	0.323	3	1	5	0.045	6	4	8	0.072
	Always	4.5	1	3		3.5	1	5		7	6	8		8	6	8		3	1	6		5	3	8		5	1	6		7	4	10	
	Only at 1	2	0	4		3	1	9										2	1	3.5		6	4	7		3.5	1.5	4.5		5.5	3	9	
	Only at 4-5	4	2	4.5		5.5	4	7		6	6	6		5	5	5		4	1	7		8	4	9		5	2	8		7.5	5	12	
Presence of bird	Never	3	1	5	0.888	5	3	7	0.840	3	1	5	0.199	5	3	7	0.005	3	1	5	0.759	5	3	8	0.388	3	1	5	0.535	6	4	9	0.744
	Always	2	2	5		4	4	7		3	3	4		9	7	13		2	1	4		6	3	7		3	2	5		5.5	3	9	
	Only at 1	3.5	2	6		3	1	7		1.5	0	3		0.5	0	1		3.5	1	5		3.5	2	9		3	1	5		6	4	8	
	Only at 4-5	3	1	4		5	2	7		1	0	3		2.5	2	5		3	1	5		6	4	8		4	2	5		6	4	9	
Presence of other animals	Never	3	1	5	0.027	5	3	7.5	0.648	3	1	5	0.424	5	3	7	0.362	3	1	5	0.036	5	2	7	0.127	3	1	5	0.484	6	4	8	0.675
	Always	1	0.5	2		4	3.5	5		5	3	6		5	5	8		2	1	4		3	2	7		3	1	4		6	3	7	
	Only at 1	3	1	5		5	3	7		2	1	4		6	3	8		3	1	5		5.5	3.5	8.5		3	2	5		6	4	10	
	Only at 4-5	2	1	5.5		6	4.5	8.5		2.5	1	4		5	3	7		4.5	2	6		5.5	3.5	9		2	1	5		6	4	9	

Med median, *P25* percentile 25, *P75* percentile 75. [a]*P* value of Kruskal–Wallis test for differences between groups

Supplementary Table 6a. Association of sample characteristics with having any pet

Variables		Has the child had any pet?								
		Never		Always		Only at 1		Only at 4-5		
		n	%	*n*	%	*n*	%	*n*	%	P^a
Family social class	Higher (I + II)	366	56.3	124	19.1	58	8.9	102	15.7	< 0.001
	Middle (III)	229	45.0	125	24.6	53	10.4	102	20.0	
	Lower (IV + V)	301	36.8	224	27.4	138	16.9	155	18.9	
Maternal education level	Up to Primary	139	30.3	146	31.8	94	20.5	80	17.4	< 0.001
	Secondary	360	42.3	212	24.9	108	12.7	172	20.2	
	University	419	58.0	131	18.1	54	7.5	118	16.3	
Paternal employment (4-5 y)	Working	835	48.7	411	24.0	137	8.0	332	19.4	0.006
	Not working	74	39.2	67	35.4	15	7.9	33	17.5	
Maternal country of origin	Spain	860	45.2	465	24.4	234	12.3	343	18.0	0.061
	Not Spain	62	42.8	27	18.6	28	19.3	28	19.3	
Paternal country of origin	Spain	852	45.0	461	24.4	237	12.5	343	18.1	0.363
	Not Spain	73	46.2	31	19.6	26	16.5	28	17.7	
Parity	0	558	49.2	249	21.9	124	10.9	204	18.0	0.001
	1	292	40.6	185	25.7	111	15.4	131	18.2	
	2+	44	36.1	40	32.8	14	11.5	24	19.7	
Paternal smoking	No	806	47.2	384	22.5	207	12.1	311	18.2	< 0.001
	Yes	103	33.4	105	34.1	43	14.0	57	18.5	
Paternal smoking (7-8 years of age)	No	581	51.1	266	23.4	89	7.8	202	17.8	0.001
	Yes	207	40.7	139	27.4	53	10.4	109	21.5	
Family type (7-8 y)	Living with father	736	49.7	353	23.8	120	8.1	272	18.4	< 0.001
	Not living with father	60	32.8	60	32.8	22	12.0	41	22.4	
Maternal anxiety history	Yes	109	37.3	82	28.1	44	15.1	57	19.5	0.038
	No	816	46.3	412	23.4	221	12.5	315	17.9	
Preterm: < 37 weeks of gestation	No	890	45.3	461	23.5	251	12.8	362	18.4	0.013
	Yes	28	35.0	31	38.8	11	13.8	10	12.5	
Child's rhinitis	No	603	46.1	330	25.2	112	8.6	263	20.1	0.109
	Yes	192	52.3	88	24.0	31	8.4	56	15.3	
Sex	Female	453	45.1	258	25.7	123	12.2	171	17.0	0.239
	Male	472	44.8	237	22.5	142	13.5	203	19.3	

	Has the child had any pet?												
	Never			Always			At age 1			At age 4-5			
	Med	P25	P75	Med	P25	P75	Med	P25	P75	Med	P25	P75	P^b
Maternal age at pregnancy	31.0	29.0	34.0	30.0	28.0	34.0	30.0	27.0	33.0	30.0	28.0	33.0	< 0.001
Weeks of breastfeeding	21.9	8.7	39.3	20.4	4.4	39.0	21.4	4.3	34.7	21.6	4.4	35.0	0.053
Child's age	7.7	7.2	7.9	7.5	7.0	7.8	7.5	6.8	7.7	7.5	6.9	7.8	< 0.001
Maternal intelligence (WAIS-III) (4-5 y)	98.8	91.5	109.8	98.8	91.5	109.8	98.8	91.5	109.8	98.8	91.5	109.8	0.852
Number of siblings (4-5)	1.0	0.0	1.0	1.0	0.0	1.0	1.0	0.0	1.0	1.0	0.0	1.0	0.186

Med median, *P25* percentile 25, *P75* percentile 75. [a]*P* values of the Chi-square test; [b]*P* values of the Kruskal–Wallis test for differences between groups

Supplementary Table 6b. Association of sample characteristics with having a dog

Variables		Presence of dog								
		Never		Always		Only at 1		Only at 4-5		
		n	%	*n*	%	*n*	%	*n*	%	*P*[a]
Family social class	Higher (I + II)	508	82.6	54	8.8	26	4.2	27	4.4	0.034
	Middle (III)	368	77.3	60	12.6	17	3.6	31	6.5	
	Lower (IV + V)	547	75.6	99	13.7	38	5.2	40	5.5	
Maternal education level	Up to primary	275	68.9	67	16.8	32	8.0	25	6.3	< 0.001
	Secondary	606	77.6	96	12.3	35	4.5	44	5.6	
	University	582	84.5	56	8.1	18	2.6	33	4.8	
Paternal employment (4-5 y)	Working	1313	79.1	184	11.1	74	4.5	88	5.3	0.148
	Not working	133	72.3	30	16.3	10	5.4	11	6.0	
Maternal country of origin	Spain	1360	77.8	208	11.9	83	4.8	96	5.5	0.514
	Not Spain	110	83.3	12	9.1	4	3.0	6	4.5	
Paternal country of origin	Spain	1356	78.1	200	11.5	83	4.8	97	5.6	0.568
	Not Spain	117	80.1	19	13.0	5	3.4	5	3.4	
Parity	0	825	78.1	132	12.5	50	4.7	49	4.6	0.096
	1	521	80.0	63	9.7	26	4.0	41	6.3	
	2+	75	70.1	19	17.8	5	4.7	8	7.5	
Paternal smoking	No	1265	80.2	166	10.5	68	4.3	78	4.9	< 0.001
	Yes	188	67.9	50	18.1	16	5.8	23	8.3	
Paternal smoking (7-8 years of age)	No	893	81.9	116	10.6	40	3.7	42	3.8	0.001
	Yes	356	73.1	61	12.5	36	7.4	34	7.0	
Family type (7-8 y)	Living with father	1142	80.3	153	10.8	64	4.5	63	4.4	0.005
	Not living with father	121	69.1	29	16.6	10	5.7	15	8.6	
Maternal anxiety history	Yes	210	78.1	37	13.8	10	3.7	12	4.5	0.522
	No	1267	78.4	183	11.3	78	4.8	89	5.5	
Preterm: < 37 weeks of gestation	No	1416	78.6	202	11.2	83	4.6	101	5.6	0.009
	Yes	52	70.3	17	23.0	4	5.4	1	1.4	
Child's rhinitis	No	994	79.3	148	11.8	53	4.2	58	4.6	0.227
	Yes	275	77.7	36	10.2	23	6.5	20	5.6	
Sex	Female	719	78.8	112	12.3	36	3.9	46	5.0	0.391
	Male	760	77.9	108	11.1	52	5.3	56	5.7	

	Presence of dog												
	Never			Always			Only at 1			Only at 4-5			
	Med	P25	P75	Med	P25	P75	Med	P25	P75	Med	P25	P75	*P*[b]
Maternal age at pregnancy	31.0	29.0	34.0	30.0	27.0	33.5	30.0	27.6	33.0	30.0	27.0	33.0	0.001
Weeks of breastfeeding	21.9	6.9	39.1	19.4	4.4	39.4	21.6	8.7	34.6	17.5	3.0	34.7	0.399
Child's age	7.6	7.0	7.8	7.6	7.3	7.8	7.5	6.8	7.7	7.6	7.3	8.0	0.059
Maternal intelligence (WAIS-III) (4-5 y)	98.8	91.5	109.8	98.8	91.5	106.2	102.5	91.5	106.2	98.8	87.8	109.8	0.435
Number of siblings (4-5)	1.0	0.0	1.0	1.0	0.0	1.0	1.0	0.0	1.0	1.0	0.0	1.0	0.311

Med median, *P25* percentile 25, *P75* percentile 75. [a]*P* values of the Chi-square test; [b]*P* value of the Kruskal–Wallis test for differences between groups

Supplementary Table 6c. Association of sample characteristics with having a cat

Variables		Presence of cat: Never		Always		Only at 1		Only at 4-5		
		n	%	*n*	%	*n*	%	*n*	%	P^a
Family social class	Higher (I + II)	550	91.5	27	4.5	14	2.3	10	1.7	0.615
	Middle (III)	426	90.1	25	5.3	11	2.3	11	2.3	
	Lower (IV + V)	652	90.7	34	4.7	11	1.5	22	3.1	
Maternal education level	Up to primary	350	88.6	23	5.8	10	2.5	12	3.0	0.336
	Secondary	706	90.7	35	4.5	14	1.8	23	3.0	
	University	619	91.8	32	4.7	14	2.1	9	1.3	
Paternal employment (4-5 y)	Working	1491	91.0	77	4.7	33	2.0	38	2.3	0.718
	Not working	162	88.5	10	5.5	5	2.7	6	3.3	
Maternal country of origin	Spain	1568	90.8	84	4.9	33	1.9	41	2.4	0.446
	Not Spain	115	87.8	8	6.1	5	3.8	3	2.3	
Paternal country of origin	Spain	1555	90.7	81	4.7	36	2.1	42	2.5	0.521
	Not Spain	132	90.4	10	6.8	2	1.4	2	1.4	
Parity	0	953	91.4	51	4.9	14	1.3	25	2.4	0.156
	1	580	90.1	29	4.5	21	3.3	14	2.2	
	2+	94	89.5	6	5.7	1	1.0	4	3.8	
Paternal smoking	No	1417	90.9	77	4.9	30	1.9	35	2.2	0.86
	Yes	244	89.4	15	5.5	6	2.2	8	2.9	
Paternal smoking (7-8 y)	No	984	91.5	49	4.6	20	1.9	22	2.0	0.052
	Yes	441	91.3	18	3.7	12	2.5	12	2.5	
Family type (7-8 y)	Living with father	1299	92.5	54	3.8	27	1.9	25	1.8	0.002
	Not living with father	145	84.3	16	9.3	5	2.9	6	3.5	
Maternal anxiety history	Yes	235	87.4	17	6.3	5	1.9	12	4.5	0.057
	No	1455	91.2	75	4.7	33	2.1	32	2.0	
Preterm: < 37 weeks of gestation	No	1618	90.9	85	4.8	36	2.0	41	2.3	0.251
	Yes	65	87.8	6	8.1	0	0.0	3	4.1	
Child's rhinitis	No	1133	91.4	53	4.3	28	2.3	25	2.0	0.616
	Yes	316	90.8	18	5.2	5	1.4	9	2.6	
Sex	Female	830	91.7	41	4.5	16	1.8	18	2.0	0.495
	Male	863	89.7	51	5.3	22	2.3	26	2.7	

	Presence of cat: Never			Always			Only at 1			Only at 4-5			
	Med	P25	P75	Med	P25	P75	Med	P25	P75	Med	P25	P75	P^b
Maternal age at pregnancy	31.0	28.0	34.0	31.0	28.5	34.0	30.0	28.0	35.0	29.0	26.0	32.5	0.059
Weeks of breastfeeding	21.9	6.4	39.0	17.1	4.3	39.4	26.1	17.1	43.6	21.6	2.0	35.0	0.266
Child's age	7.6	7.0	7.8	7.5	6.9	7.8	7.2	6.4	7.8	7.6	7.4	8.1	0.029
Maternal intelligence (WAIS-III) (4-5 y)	98.8	91.5	109.8	102.5	95.1	113.5	102.5	95.1	113.5	102.5	91.5	113.5	0.255
Number of siblings (4-5)	1.0	0.0	1.0	1.0	0.0	1.0	1.0	1.0	1.0	1.0	0.0	1.0	0.190

Med median, *P25* percentile 25, *P75* percentile 75. [a]*P* values of the Chi-square test; [b]*P* value of the Kruskal–Wallis test for differences between groups

Supplementary Table 6d. Association of sample characteristics with having a bird

Variables		Presence of bird								
		Never		Always		Only at 1		Only at 4-5		
		n	%	*n*	%	*n*	%	*n*	%	*P*[a]
Family social class	Higher (I + II)	553	91.7	11	1.8	14	2.3	25	4.1	< 0.001
	Middle (III)	397	84.3	21	4.5	17	3.6	36	7.6	
	Lower (IV + V)	565	78.5	41	5.7	41	5.7	73	10.1	
Maternal education level	Up to Primary	300	75.9	29	7.3	20	5.1	46	11.6	< 0.001
	Secondary	650	83.5	34	4.4	36	4.6	58	7.5	
	University	610	90.4	12	1.8	21	3.1	32	4.7	
Paternal employment (4-5 y)	Working	1395	85.1	61	3.7	66	4.0	117	7.1	0.263
	Not working	150	82.0	12	6.6	6	3.3	15	8.2	
Maternal country of origin	Spain	1465	84.8	66	3.8	71	4.1	126	7.3	0.535
	Not Spain	105	80.8	8	6.2	6	4.6	11	8.5	
Paternal country of origin	Spain	1456	84.9	68	4.0	67	3.9	124	7.2	0.158
	Not Spain	117	80.1	5	3.4	11	7.5	13	8.9	
Parity	0	903	86.7	26	2.5	39	3.7	74	7.1	0.006
	1	527	81.6	39	6.0	28	4.3	52	8.0	
	2+	84	80.0	8	7.6	5	4.8	8	7.6	
Paternal smoking	No	1333	85.5	63	4.0	53	3.4	110	7.1	< 0.001
	Yes	212	77.4	12	4.4	23	8.4	27	9.9	
Paternal smoking (7-8 y)	No	920	85.5	42	3.9	41	3.8	73	6.8	0.680
	Yes	409	84.7	15	3.1	19	3.9	40	8.3	
Family type (7-8 y)	Living with father	1198	85.1	52	3.7	54	3.8	103	7.3	0.742
	Not living with father	142	83.0	9	5.3	6	3.5	14	8.2	
Maternal anxiety history	Yes	213	79.5	11	4.1	17	6.3	27	10.1	0.055
	No	1362	85.3	64	4.0	61	3.8	110	6.9	
Preterm: < 37 weeks of gestation	No	1506	84.5	72	4.0	73	4.1	131	7.4	0.932
	Yes	60	82.2	3	4.1	4	5.5	6	8.2	
Child's rhinitis	No	1048	84.6	47	3.8	50	4.0	94	7.6	0.790
	Yes	302	86.5	13	3.7	11	3.2	23	6.6	
Sex	Female	759	83.5	40	4.4	45	5.0	65	7.2	0.350
	Male	818	85.3	36	3.8	33	3.4	72	7.5	

	Presence of bird												
	Never			Always			Only at 1			Only at 4-5			
	Med	P25	P75	Med	P25	P75	Med	P25	P75	Med	P25	P75	*P*[b]
Maternal age at pregnancy	31.0	29.0	34.0	30.0	28.0	34.0	31.0	27.0	33.0	30.0	27.0	33.0	0.050
Weeks of breastfeeding	21.9	6.4	39.0	21.9	4.3	47.9	17.1	2.0	32.3	19.1	6.4	39.0	0.171
Child's age	7.6	7.0	7.9	7.5	7.2	7.7	7.5	7.1	7.6	7.5	6.9	7.7	< 0.001
Maternal intelligence (WAIS-III) (4-5 y)	98.8	91.5	109.8	97.0	84.1	109.8	102.5	95.1	109.8	95.1	84.1	106.2	0.013
Number of siblings (4-5)	1.0	0.0	1.0	1.0	1.0	1.0	1.0	0.0	1.0	1.0	0.0	1.0	0.149

Med median, *P25* percentile 25, *P75* percentile 75. [a]*P* values of the Chi-square test; [b]*P* value of the Kruskal–Wallis test for differences between groups

Supplementary Table 6e. Association of sample characteristics with having any other animal

Variables		Presence of other animals: Never		Always		Only at 1		Only at 4-5		
		n	%	*n*	%	*n*	%	*n*	%	*P*[a]
Family social class	Higher (I + II)	463	76.3	31	5.1	85	14.0	28	4.6	0.022
	Middle (III)	333	69.4	25	5.2	96	20.0	26	5.4	
	Lower (IV + V)	492	68.0	54	7.5	137	18.9	41	5.7	
Maternal education level	Up to Primary	260	65.2	32	8.0	76	19.0	31	7.8	< 0.001
	Secondary	531	67.5	51	6.5	165	21.0	40	5.1	
	University	536	78.9	33	4.9	84	12.4	26	3.8	
Paternal employment (4-5 y)	Working	1189	71.8	100	6.0	282	17.0	84	5.1	0.645
	Not working	125	67.6	13	7.0	35	18.9	12	6.5	
Maternal country of origin	Spain	1238	71.0	111	6.4	309	17.7	86	4.9	0.146
	Not Spain	99	75.6	4	3.1	18	13.7	10	7.6	
Paternal country of origin	Spain	1227	70.8	109	6.3	303	17.5	93	5.4	0.273
	Not Spain	113	77.4	6	4.1	23	15.8	4	2.7	
Parity	0	794	75.4	44	4.2	184	17.5	31	2.9	< 0.001
	1	424	65.1	59	9.1	108	16.6	60	9.2	
	2+	69	65.1	7	6.6	26	24.5	4	3.8	
Paternal smoking	No	1135	72.3	99	6.3	267	17.0	69	4.4	0.003
	Yes	178	63.6	17	6.1	61	21.8	24	8.6	
Paternal smoking (7-8 y)	No	792	72.7	62	5.7	182	16.7	53	4.9	0.001
	Yes	324	66.3	39	8.0	96	19.6	30	6.1	
Family type (7-8 y)	Living with father	1019	71.6	93	6.5	241	16.9	71	5.0	0.007
	Not living with father	107	62.2	8	4.7	41	23.8	16	9.3	
Maternal anxiety history	Yes	171	63.6	19	7.1	59	21.9	20	7.4	0.023
	No	1168	72.4	97	6.0	270	16.7	78	4.8	
Preterm: < 37 weeks of gestation	No	1276	71.0	108	6.0	319	17.8	94	5.2	0.274
	Yes	54	72.0	8	10.7	9	12.0	4	5.3	
Child's rhinitis	No	870	69.4	76	6.1	237	18.9	70	5.6	0.203
	Yes	261	73.7	25	7.1	51	14.4	17	4.8	
Sex	Female	642	70.1	60	6.6	166	18.1	48	5.2	0.806
	Male	698	72.0	57	5.9	164	16.9	50	5.2	

	Presence of other animals: Never			Always			Only at 1			Only at 4-5			
	Med	P25	P75	Med	P25	P75	Med	P25	P75	Med	P25	P75	*P*[b]
Maternal age at pregnancy	31.0	29.0	34.0	31.0	28.0	34.0	30.0	27.0	33.0	31.0	28.0	34.0	0.024
Weeks of breastfeeding	21.8	6.4	39.1	21.9	4.9	34.8	21.9	6.3	39.0	19.4	4.4	37.0	0.941
Child's age	7.7	7.1	7.9	7.4	6.7	7.6	7.4	6.8	7.8	7.6	7.1	7.8	<0.001
Maternal intelligence (WAIS-III) (4-5 y)	98.8	87.8	109.8	102.5	91.5	113.5	98.8	91.5	109.8	98.8	91.5	106.2	0.274
Number of siblings (4-5)	1.0	0.0	1.0	1.0	1.0	1.0	1.0	0.0	1.0	1.0	1.0	1.0	0.002

Med median, *P25* percentile 25, *P75* percentile 75. [a]*P* values of the Chi-square test; [b]*P* value of the Kruskal–Wallis test for differences between groups
